# A way to understand idiopathic senescence and apoptosis in primary glioblastoma cells – possible approaches to circumvent these phenomena

**DOI:** 10.1186/s12885-019-6130-2

**Published:** 2019-09-14

**Authors:** Karolina Janik, Cezary Treda, Aneta Wlodarczyk, Joanna Peciak, Kamila Rosiak, Jolanta Zieba, Dagmara Grot, Adrianna Rutkowska, Roza Pawlowska, Waldemar Och, Piotr Rieske, Ewelina Stoczynska-Fidelus

**Affiliations:** 1 Department of Tumor Biology, Medical University of Lodz, Chair of Medical Biology, Zeligowskiego 7/9, 90-752 Lodz, Poland; 2 Department of Research and Development, Celther Polska Ltd., Milionowa 23, 93-193 Lodz, Poland; 3 Department of Bioorganic Chemistry, Centre of Molecular and Macromolecular Studies, Polish Academy of Sciences, Sienkiewicza 112, 90-363 Lodz, Poland; 4 Clinical Department of Neurosurgery, The Voivodal Specialistic Hospital in Olsztyn, Zolnierska 18, 10-561 Olsztyn, Poland

**Keywords:** Senescence, Apoptosis, Glioblastoma, Immortalization, In vivo model

## Abstract

**Background:**

Glioblastoma (GB) is considered one of the most lethal tumors. Extensive research at the molecular level may enable to gain more profound insight into its biology and thus, facilitate development and testing of new therapeutic approaches. Unfortunately, stable glioblastoma cell lines do not reflect highly heterogeneous nature of this tumor, while its primary cultures are difficult to maintain in vitro. We previously reported that senescence is one of the major mechanisms responsible for primary GB cells stabilization failure, to a lesser extent accompanied by apoptosis and mitotic catastrophe-related cell death.

**Methods:**

We made an attempt to circumvent difficulties with glioblastoma primary cultures by testing 3 different approaches aimed to prolong their in vitro maintenance, on a model of 10 patient-derived tumor specimens.

**Results:**

Two out of ten analyzed GB specimens were successfully stabilized, regardless of culture approach applied. Importantly, cells transduced with immortalizing factors or cultured in neural stem cell-like conditions were still undergoing senescence/apoptosis. Sequential in vivo/in vitro cultivation turned out to be the most effective, however, it only enabled to propagate cells with preserved molecular profile up to 3^rd^ mice transfer. Nevertheless, it was the only method that impeded these phenomena long enough to provide sufficient amount of material for in vitro*/*in vivo targeted analyses. Interestingly, our data additionally demonstrated that some subpopulations of several stabilized GB cell lines undergo idiopathic senescence, however, it is counterbalanced by simultaneous proliferation of other cell subpopulations.

**Conclusions:**

In the majority of primary glioma cultures, there has to be an imbalance towards apoptosis and senescence, following few weeks of rapid proliferation. Our results indicate that it has to be associated with the mechanisms other than maintenance of glioblastoma stem cells or dependence on proteins controlling cell cycle.

## Background

Limitless proliferative potential is thought to be one of the most characteristic features of cancer cells [1], however, it is not fully reflected in vitro, as maintenance of primary cancer cells in culture is highly limited [2]. Glioblastoma (GB) is one of the most aggressive tumors of the central nervous system, associated with poor prognosis and lack of effective treatment. Although extensively explored, stable GB cell lines, such as U87-MG or T98G, are rather more homogenous populations than an accurate representation of molecularly diverse glioma cells observed in vivo [3]. The availability of cell lines characterized with oncogenes amplifications, *EGFR*^*vIII*^ or *IDH1*^*R132H*^ mutations, commonly observed in this tumor type is severely limited [4, 5], while primary GB cultures tend to be difficult to establish.

Senescence is one of the mechanisms associated with culturing difficulties of primary cancer cells and it has already been described in various cancer cell types [6, 7]. We previously reported that GB cells undergo senescence in vitro very early in culture (2^nd^ – 3^rd^ passage) and avoid stabilization attempts [4]. Other accompanying phenomena include spontaneous or idiopathic apoptosis and cell death resulting from mitotic catastrophe [4], but these have not been profoundly analyzed so far.

Recent analysis of culturing methods of primary GB cells indicates that there is plethora of published protocols, differing in culture medium, plate coating or culture type [4, 8–14]. Therefore, there is a lack of standardized and unified method of establishment and maintenance of such cultures. Due to this fact it is difficult to compare establishment efficiency between different laboratories, as obtained results are often even contradictory [15]. To further complicate this issue, it is worth to emphasize that glioblastoma is molecularly classified into four subtypes [16], and each may require different culture conditions or establishment approach. Nevertheless, it remains debatable whether culturing inconsistencies actually depend on applied conditions, or rather on molecular profile of tumor cells. Without the precise molecular characterization it is not clear what type of cells (tumor or normal cells infiltrating tumor mass) actually preserves in long-term culture. Currently there is a tendency to limit the number of reported passages and restrict molecular identification of cells to tissue samples, with no molecular data of cultured cells available [12, 16].

As the exact mechanism hindering stabilization of proliferating GB cells remains elusive, in this paper we analyzed three different approaches of glioblastoma cells culturing in an attempt to try to understand and circumvent senescence and cell death, hence, prolonging in vitro maintenance of cells with preserved phenotype and genotype. Determination of the most optimal approach will not only enable to employ primary GB cultures for complex in vitro analyses, but also possibly provide an insight into the mechanisms underlying culturing difficulties.

## Methods

### Tissue samples

Tissue samples were obtained from 10 patients diagnosed with glioblastoma, IDH-wild type (8 cases) and IDH-mutant (2 cases) according to the latest World Health Organization criteria [17], and treated at the Clinical Department of Neurosurgery, Voivodal Specialistic Hospital in Olsztyn. All samples were collected using the protocol approved by the Bioethical Committee of the Medical University of Lodz (Approval No. RNN/234/17/KE). Written informed consent was obtained from all patients and their data were processed and stored according to the principles expressed in the Declaration of Helsinki. Tumor specimens were shipped in 1x Hank’s Balanced Salt Solution (HBSS; Biowest) and isolation of cells started no longer than 5 h following surgical operation. The surgical samples were rendered anonymous and coded.

### Cell cultures

#### Primary glioblastoma cultures – adherent conditions

All fresh glioblastoma tumor samples were initially washed twice with 1x HBSS and centrifuged (80 xg, 90 s). Following transfer onto 10 cm culture dishes, specimens were cut into fragments <1 mm^3^ and part of material was further enzymatically dispersed (37 °C, 45 min – 1.5 h, depending on the fragment size) with collagenase type IV (200 U/mL, Sigma-Aldrich), dispase (1 U/mL; STEMCELL Technologies) and DNaseI (3 μg/mL; STEMCELL Technologies). Following filtration through 70 μm cell strainer cells were centrifuged (80 xg, 90 s) and subsequently suspended in Astrocyte Growth Medium (AGM; Lonza) for adherent GB cultures or Pericyte Growth Medium (PromoCell) for pericyte cultures. Depending on proliferation rate, cells were passaged with StemPro Accutase (Gibco) to a new culture dish every 7–14 days. For further immunofluorescence analyses, cells were passaged onto 4 well plates with coverslips and left to adhere.

#### Primary glioblastoma cultures – neural stem cell-like conditions

Another part of material, obtained from 5/10 tumor specimens following mechanical disintegration (as described above), was further established according to the protocol developed by Xie et al. [16]. Briefly, minced tumor tissue was incubated in 1:1 mixture of StemPro Accutase and TrypLE (10 min, 37 °C; Invitrogen). Following filtration through 70 μm cell strainer cells were centrifuged (80 xg, 90 s) and subsequently suspended in 1:1 mixture of Neurobasal medium (Life Technologies) and DMEM/F12 (Biowest), supplemented with N2, B27, Antimitotic-antimycotic, Glutamax (1x; all Life Technologies), NEAA (1x; Biowest), bFGF (40 ng/mL, Peprotech) and EGF (5 ng/mL, Peprotech). Approximately 5–7 days later, formed spheres were treated with StemPro Accutase, washed with culture medium and seeded onto 6-well plates previously coated with poly-l-ornithine (10 μg/mL; 2 h, RT; Sigma Aldrich) and further with mouse laminin (10 μg/mL; 30 min, 37 °C; Sigma Aldrich). Depending on proliferation rate, such adherent cultures were passaged with StemPro Accutase to a new culture dish every 7–14 days. For further immunofluorescence analyses, spheres were passaged onto 4 well plates with coverslips and left to adhere and let cells migrate out of sphere.

#### Stable cell lines

DK-MG cells (DSMZ) were cultured in RPMI 1640 medium (Gibco), U87-MG, T98G (both ATCC), A9 cell line (established at the Department of Tumor Biology with the use of the protocol described above for the adherent conditions) were cultured in MEM (Gibco), while NIH/3T3, NTERA-2 and HEK-293 T cells (all ATCC) in DMEM (Biowest). Each medium was supplemented with 10% FBS, 1% penicillin/streptomycin and 0.2% gentamicin (all Biowest). Cells were cultured in 5% CO_2_ and passaged with trypsin-EDTA (0.05% trypsin; Biowest).

### Lentivirus preparation

RNA from neural cell line NTERA-2 was isolated using AllPrep DNA/RNA Mini Kit and reverse transcribed using QuantiTect Rev. Transcription Kit (both Qiagen). *SV40*, *BMI-1* and *hEST2* genes were amplified using Q5® Hot Start High-Fidelity DNA Polymerase (NEB) and Gateway® specific primers (Additional file 1: Table S1). Gateway® BP Clonase® II Enzyme mix (Life Technologies) was used to introduce each gene to pENTR/Zeo vector. Lentiviral particles were generated as previously described [18]. Briefly, HEK293T cells were seeded at a density of 5× 10^6^ cells per 10 cm dish and 12–16 h after the initial plating, culture media were discarded and replaced with 12 mL of fresh ones. HEK293T cells were simultaneously transiently transfected using linear polyethylenimine (PEI, 25 kDa; Polysciences) at 2:1 PEI to DNA ratio, with packaging plasmid (pLV-HELP), envelope plasmid (pLV-iVSV-G, for pseudotyping viral particles with pantropic VSV-G protein; both InvivoGen) and the transfer vector carrying the gene of interest. One day after transfection, culture media were replaced with fresh ones, and additionally supplemented with 10 mM HEPES buffer (Gibco). Cell supernatants containing lentiviral particles were collected after 24 and 48 h. Cellular debris was removed from the supernatants by centrifugation (2000 ×g, 5 min) and filtration through a 0.45 μm PES (low protein binding) filter. Afterwards, lentiviral particles were concentrated by ultrafiltration with 100 kDa cut-off Amicon centrifugal filter (EMD Millipore), aliquoted and stored at − 80 °C.

### Transduction of glioblastoma cells

Glioblastoma neurospheres obtained from five patients with the protocol by Xie et al. were centrifuged (80 xg, 90 s) and suspended in StemPro Accutase. After 5 min remaining spheres were disrupted mechanically, cells were washed twice with ice cold PBS, counted and seeded onto 24-well plate (2.5 × 10^4^ cells/well) in AGM medium. After 2 days wells were checked for the attached cells, and fresh medium was added. To each well 0.2 μg/mL of polybrene with lentivirus (MOI = 10) was added. Two days later medium was renewed and puromycin selection of transduced cells was conducted. Cells transduced with empty lentivirus and lacking lentivirus served as negative controls.

### In vivo study

All studies concerning animals were conducted in accordance with the approval of the Bioethical Committee of the Medical University of Lodz (Approval No. 76/LB586/2011). Fresh neurospheres (up to 7 days post seeding) were centrifuged (80 xg, 90 s), incubated in StemPro Accutase (5 min, RT) and mechanically disrupted. Cells were washed twice with PBS and counted. Half of cells was mixed with Matrigel, while the other half was suspended in PBS and >10^4^ cells/mouse were injected subcutaneously into 5–6 week old Crl:SHO-PrkdcscidHrhr mice (Animalab) [2]. Tumor volume was monitored by a measurement according to the following formula: 0.5xy^2^ [3]. After 6–8 weeks mice were sacrificed, tumor was carefully excised, washed and transferred onto 10 cm culture dish. Following mechanical disintegration into fragments <1 mm^3^ and enzymatic digestion with collagenase type IV (200 U/mL, 37 °C for 6 h), dispase (1 U/mL, 30 min, 37 °C) and DNaseI (3 μg/mL), cells were filtered using a 70 μm cell strainer, washed twice with 1x HBSS and centrifuged (80 xg, 90 s). Finally, cells were suspended in PBS, counted, mixed with Matrigel and once again injected as described aboveor transferred onto new culture vessels according to the protocol by Xie et al. The procedure was conducted for three out of ten tumor samples (GB6, GB8, GB9) till molecular analyses revealed lack of tumor cells in the material or molecular incompatibility when compared to tissue of origin. Three transfers were conducted for GB6 and GB9 tumors, while two transfers for GB8. In total, analyses were conducted on 17 mice (one mouse per T1, two mice per T2 and 4 mice per T3). Mice were euthanized by intraperitoneal injection of a ketamine/xylazine solution [ketamine (50 mg/kg bw) and xylazine (5 mg/kg bw)]. Injections were performed using 25G needles with maximum administration volume not exceeding 125 μl. Following anesthesia, the tumor material was collected and mice were sacrificed by cervical dislocation.

### Multiplex ligation-dependent probe amplification (MLPA)

In order to identify the origin of analyzed cells in tissue samples as well as throughout the culture course, MLPA reactions were performed using P294 Tumor Loss or P175 Tumor Gain (in some cases also P105-D1 Glioma-2) probe mixes and dedicated kits (MRC-Holland), according to the manufacturer’s protocol, as described previously [6]. Fragments were separated by capillary electrophoresis using ABI 3130 Genetic Analyzer and comparative analyses were performed using the latest version of Coffalyzer. Net (MRC-Holland). For each gene, the resultant ratio was calculated and interpreted as normal copy number (0.7–1.3), deletion of one allele (0.35–0.65), deletion of both alleles (0), gain of one allele (e.g. trisomy; 1.35–1.55) and gain of more than one allele (1.6–2.2), while the value in range of 0.1–0.3 was considered to result from the analysis of heterogeneous material in which deletion was detected.

### Immunofluorescence analyses

For immunocytochemical stainings cells were fixed in cold 4% paraformaldehyde (PFA) in PBS (14 min, RT) and permeabilized with 0.1% Triton X-100 (10 min, RT). Nonspecific binding sites were blocked by incubation with 2% donkey serum (Sigma Aldrich) in PBS for 1 h. For double or triple immunolabeling, fixed cells were subsequently incubated with the appropriate primary antibodies (1 h, RT; Additional file 2: Table S2) and further visualized by simultaneous incubation with a combination of species-specific fluorochrome-conjugated secondary antibodies (1 h, RT; Additional file 2: Table S2). Control samples were incubated with the secondary antibodies alone and were otherwise processed identically. The slides were mounted with ProLong® Gold Antifade Reagent with DAPI (Invitrogen), coverslipped and examined using OPTA-TECH MN 800 fluorescence microscope. DAPI staining also constituted an additional method for apoptosis detection, and, together with staining for acetylated alpha-tubulin and phosphorylated histone H3 for mitotic catastrophe detection. The percentage of apoptotic nuclei as well as the percentage of cells following mitotic catastrophe or undergoing abnormal mitoses (demonstrating spindle apparatus/chromosomal misalignments) was calculated from at least ten visual fields, comprising of at least 200 cells per case.

### Senescence associated (SA)-β-galactosidase assay

SA-β-Gal staining was performed using Senescence Cells Histochemical Staining Kit (Sigma Aldrich). Firstly, cells were washed thrice with PBS, fixed with cold 4% PFA for 10 min and then washed two times with PBS for 5 min. Next, cells were incubated with freshly prepared staining mixture (12 h, 37 °C, no CO_2_). Following incubation cells were washed twice with PBS for 5 min and photographed using Olympus CKX41 light microscope. The percentage of positively stained cells was subsequently calculated from at least ten visual fields comprising of at least 200 cells per case.

### Bromodeoxyuridine (BrdU) incorporation assay

The detection of proliferating cells was conducted as previously described [19]. Briefly, 10 μM BrdU was added to the cultures and after 24 - 120 h immunocytochemical staining for other markers was performed as described above. Subsequently, cells were post-fixed in 4% PFA and once again permeabilized with 0.1% Triton X-100 (10 min, RT). Non-specific binding sites were blocked by the incubation with 2% donkey serum in PBS for 30 min. After blocking, the cells were treated with 2 N HCl (40 min, 37 °C) and then with 0.1 M borate buffer (pH = 8.5, 12 min, RT). Then, cells were incubated with anti-BrdU antibody (1:500, 1 h; Sigma-Aldrich), washed with PBS and incubated with the appropriate secondary antibodies (1 h, RT in dark). Finally, cells were mounted with ProLong® Gold Antifade Reagent (Molecular Probes), coverslipped and examined using OPTA-TECH MN 800 fluorescence microscope. For each analysis 200 nuclei were examined.

### Real-time observations and image analyses

Glioblastoma cells maintained in adherent conditions (either standard adherent culture or NSC-like conditions) were seeded onto 6-well plates and subjected to real-time in vitro observations using JuLI FL live cell analyzer (NanoEntek). Five images per well were taken in bright light in 24 h intervals for up to 120 h of overall observation period and cell number was calculated in order to assess proliferation and apoptosis rates. Phenotype of analyzed cells was also considered to evaluate the occurrence of senescence or mitotic catastrophe. Moreover, to better analyze apoptosis, a synthetic Caspase 3/7 reporter was used (CellEvent™ Caspase 3/7 Green; Life Technologies). Cells were incubated with the staining solution according to manufacturer’s guidelines for 48 h and analyzed using JuLI FL analyzer. The average percentage of apoptotic cells was calculated by analyzing at least 200 cells per case.

### Other molecular analyses – reverse-transcription real-time PCR, sanger sequencing and fluorescence in situ hybridization (FISH)

DNA and RNA isolation, reverse transcription and real-time PCR reactions were performed in triplicates as described previously [20]. *TBP* gene was used for normalization of *EGFR*^*WT*^ and *EGFR*^*vIII*^ expression at mRNA level and normalized relative expression levels of target genes were calculated using the method described by Pfaffl et al. [21, 22], while *RPP25* gene was used for normalization at DNA level. To determine the copy number of *EGFR*^*vIII*^ (calculated as a difference between *EGFR*^*WT*^ and total EGFR) and *EGFR*^*WT*^ genes Real-time PCR analyses at DNA level were performed. Primer sequences used for amplification of *EGFR*^*WT*^ gene at DNA level were as follows: 5′-CTCACGCAGTTGGGCACTTT-3′, 5′-CCACCTCACAGTTATTGAACATCCT-3′ while 5′-CACACCCCTGACTCTCCACT-3′, 5′-GAGACAATCCTGTGAGCTTGG-3′ sequences were used to amplify total *EGFR* gene. The following specific primers were used for amplification of *RPP25* gene: 5′-GGGAGATGCGGAAGAATGT-3′, 5′-CCTCCAGTCAGCCACAGAA-3′. Results around 2 were interpreted as 2 gene copy number, values below 2 were considered deletion, while above 2 amplification. Sanger sequencing of *IDH1* and *TP53* genes as well as FISH analysis for *EGFR* gene were performed as described previously [4, 5].

### Statistical analyses

Statistical significance for Real-time PCR results was determined by two-way ANOVA analysis with post-analysis Bonferroni’s multiple comparisons test. Results were considered statistically significant at *p* < 0.05. Comparison of spontaneous in vitro senescence in primary GB cultures stabilized in our department and commercially available GB stable cancer cell lines was calculated based on at least 200 cells per case by paired Student’s t-test with data statistically significant at *p* < 0.05, *p* < 0.01 and *p* < 0.005. Paired Student’s t-test was also used to assess the significance of the differences in the percentage of spontaneously senescent (by means of SA-β-Gal assay), apoptotic (using in vitro real-time caspase assay), proliferating (during 5 days incubation with BrdU), as well as other cells in various culture conditions and passages, with *p* < 0.05 considered statistically significant. The average percentage of cells with particular feature was calculated by analyzing at least 200 cells per case.

## Results

### Molecular characteristics of analyzed GB samples fluctuates throughout the culture course

**Fig. 1:**
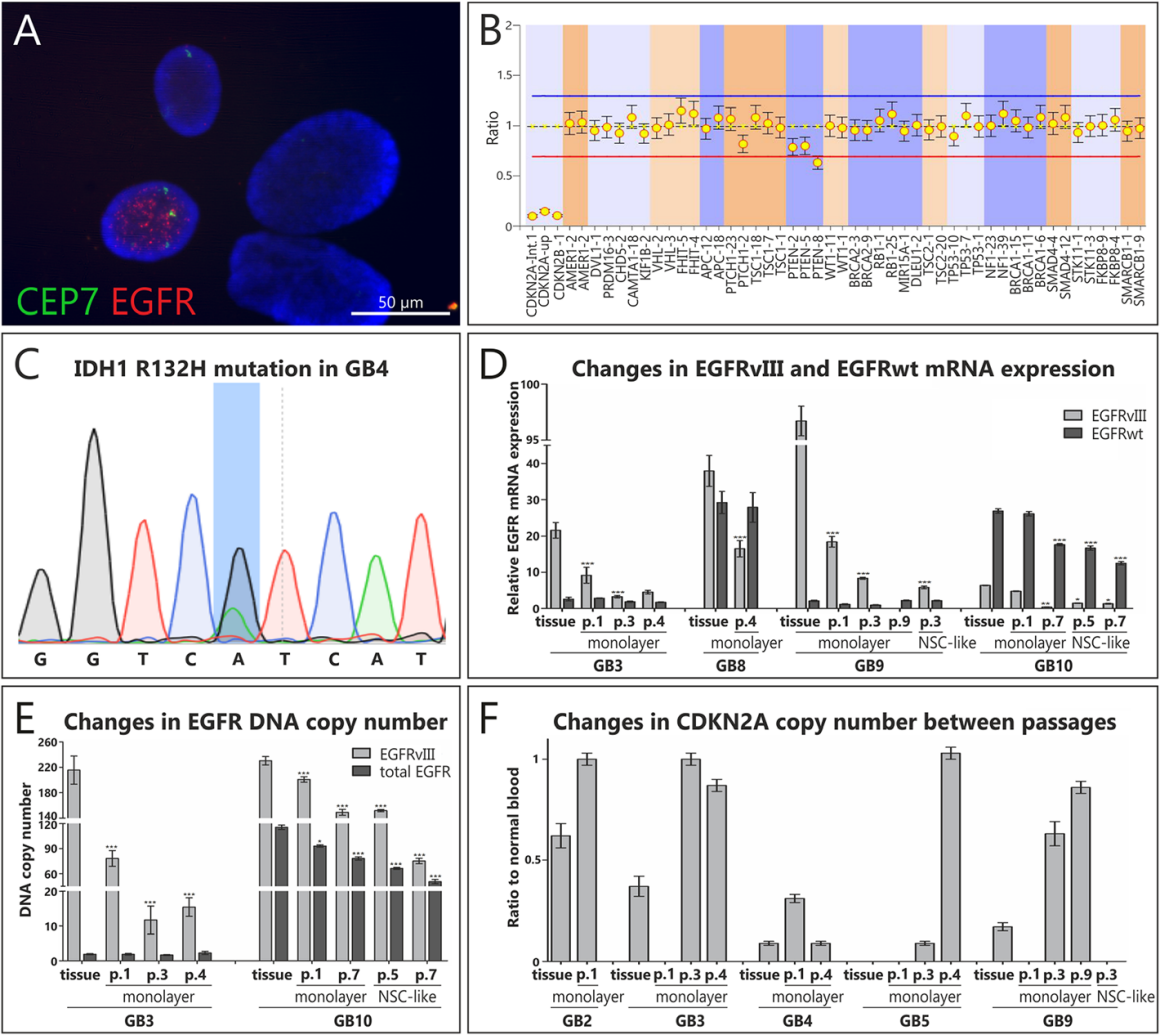
Monitoring of the stability of molecular alterations in GB cell cultures. **a-c** Representative figures showing results of conducted molecular analyses: FISH result presenting chromosome 7 trisomy and *EGFR* amplification in single cells of GB7 in passage 1 of monolayer culture (**a**); MLPA analysis showing *CDKN2A* deletion in GB5 in passage 3 of monolayer conditions (**b**); *IDH1* sequencing of GB4 – the line marks the mutated nucleotide in codon 132 (R132H) (**c**); Changes in *CDKN2A* status (**d**), EGFR^vIII^ and EGFR^WT^ DNA copy number (**e**) and *EGFR*^*WT*^ and *EGFR*^*vIII*^ mRNA expression (**f**) in the culture course of glioblastoma cells in two analyzed culture conditions. EGFR probe (red signals); CEP 7 control probe – centromere of chromosome 7, enabling to show the number of chromosomes 7 (green signals). For MLPA results error bars indicate SD, while for Real-time PCR SEM. Statistical significance for Real-time PCR results was determined by two-way ANOVA analysis with post-analysis Bonferroni’s multiple comparisons test to compare each sample to the adequate frozen sample. Results were considered statistically significant at * *p* < 0.05; ** *p* < 0.01; *** *p* < 0.005

All analyzed GB tissue specimens were initially molecularly characterized, in order to verify their neoplastic origin using various molecular techniques, such as FISH, MLPA, Sanger sequencing for *TP53* and *IDH1* status and Real-time PCR for the expression of *EGFR*^*WT*^ and *EGFR*^*vIII*^ as well as Real-time PCR at DNA level for *EGFR*^*WT/vIII*^ copy number (Tab. 1; Additional file 3: Table S3). These analyses were also conducted further in the culture course in both culture conditions tested, enabling to monitor changes in molecular alterations (Fig. 1a-f, Additional file 3: Table S3). Apart from two GB cases (GB7 and GB10) that stabilized regardless of culture conditions applied, the majority of analyzed highly heterogeneous glioblastoma cells failed to do so (no molecular changes were detected in later passages, e.g. increase in the copy number of *CDKN2A* to the normal cell level in GB5 cultured in monolayer conditions, indicating that only normal cells remained in culture as early as in passage 4; Fig. 1f; Additional file 3: Table S3). Interestingly, it is possible that some particular cell populations characterized with a specific molecular prolife were more prone to adaptation to in vitro conditions, as in case of two stabilized cases (GB7 and GB10) we detected a deletion of PTEN allele further in the culture course (Additional file 3: Table S3).

**Table 1:** Sample description and molecular alterations detected in all analyzed tumor specimens and corresponding cultures

	Patient data	MLPA		Other molecular analyses IDH1, TP53 (seq) and EGFR (FISH, RT PCR) status	In vitro culturing		Immortalization	In vivo culturing
	Sex	Gain	Del		Monolayer	NSC-like		
GB1	M	EGFR, MDM4, PDGFRA, KIT, KDR, MET	CDKN2A*, CDKN2B*, AMER1*, RET*, PTEN*	EGFRvIII amplification	Yes	N/A	N/A	N/A
GB2	F	EGFR, MDM, MET, AURKA, BRAF	CDKN2A*, PTEN*	IDH1 – NA TP53 – NA EGFRvIII amplification in single cells	Yes	N/A	N/A	N/A
GB3	M	EGFR, MET, PTEN	CDKN2A*	IDH1 p.R132H TP53 p.P190L EGFRvIII amplification	Yes	N/A	tested	N/A
GB4	F	CCND1, BRAF, AURKA	CDKN2A	IDH1 p.R132H	Yes	N/A	N/A	N/A
GB5	F	PDGFRA, KIT, KDR, NFKB1, CDK4	CDKN2A, CDKN2B, MDM2	–	Yes	N/A	N/A	N/A
GB6	F	NFKB1, PTEN	MDM2	TP53 p.P190L	Yes	Yes	tested	Yes
GB7 stabilized	F	EGFR, NFKB1A	MDM2*	TP53 p.Y205H polysomy, EGFRvIII amplification in single cells	stabilized^+^	stabilized^+^	tested	N/A
GB8	M	EGFR, NFKB1A, PTEN	MDM2*	EGFRvIII amplification in single cells	Yes	Yes	tested	Yes
GB9	M	EGFR, MDM2, CDK4	CDKN2A, PTEN*	TP53 p.Y234* EGFRvIII amplification in single cells	Yes	Yes	tested	Yes
GB10 stabilized	M	EGFR, CDK4, MDM2, BRAF, PDGFRA	RET*	EGFRvIII amplification	stabilized^+^	stabilized^+^	N/A	N/A

*F* Female, *M* Male; *deletion of one allele; “-”not detected; *N/A* Not analyzed; seq – Sanger sequencing/IHC for *IDH1* codon 132; *TP53* exons 4–8; ^+^tumors that stabilize in NSC-like conditions tend to stabilize irrespective to culture approach; detailed data shown in Additional file 3: Table S3

### Culturing of glioblastoma cells in monolayer or NSC-like conditions is not sufficient to ensure culture maintenance

**Fig. 2:**
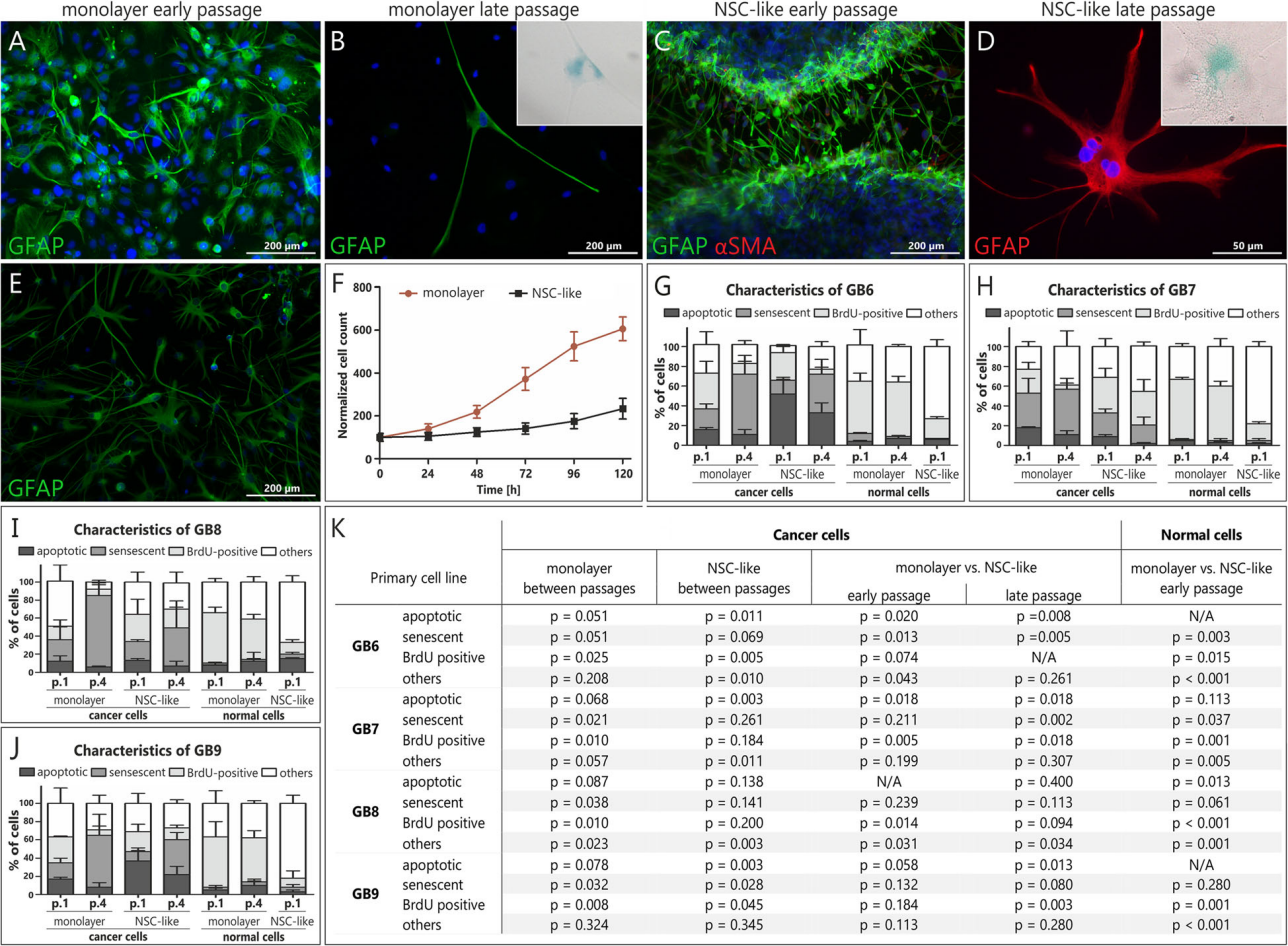
The comparison of NSC-like and classical monolayer conditions for culturing of primary glioblastoma cells. Both approaches did not enable to maintain long-term culture of GB cells and to avoid senescence/apoptosis; **a-e** Representative pictures showing results of the immunocytochemical analyses; **a** Early passage in monolayer serum conditions showing GFAP (+) GB cells; **b** Single GFAP (+), SA-β-Gal (+) GB cells from monolayer GB9 culture (intermediate passage) surrounded by cells negative for both these markers; **c** Early fresh GB9 neuropshere transferred onto poly-l-ornithine/laminin-coated dish, with the majority of GFAP (+) cells migrating from the neurosphere and only several αSMA (+) cells; **d** In late passage only large, flat and SA-β-Gal (+) cells with features of mitotic catastrophe maintained in culture; Inset boxes in B and D show higher magnifications of senescent (SA-β-Gal-positive) cells; **e** Intermediate passage of GB9 in NSC-like conditions – only GFAP (+) cells maintained in culture, but there is a lack of cells in mitosis; **f** Reduced proliferation rate of GB8 cells under NSC-like conditions vs. adherent monolayer culture; **g-j** The percentage of spontaneously senescent (as determined by means of SA-β-Gal assay), apoptotic (assessed using in vitro real-time caspase assay after 48 h of incubation with Caspase 3/7 synthetic reporter solution), proliferating (analyzed during 5 days incubation with BrdU), as well as other cells in various culture conditions and passages. The characteristics of cells in different culture conditions is depicted separately. The average percentage of cells with a particular feature was calculated by analyzing at least 200 cells per case from indicated passages of GB6-GB9 cultures. Error bars indicate SEM. **k** Statistical significance was calculated by paired Student’s t-test with *p* < 0.05 considered statistically significant

Glioblastoma cells cultured in standard monolayer conditions in early passages tend to initially, at least in part, reflect both, genotype and phenotype (*EGFR*^*WT*^ and *EGFR*^*vIII*^ expression, *CDKN2A* copy number and expression of GFAP – a specific marker for astrocytes; Fig. 1d-e; Additional file 3: Table S3), as well as heterogeneity (presence of normal GFAP-negative cell populations) of the corresponding tissue specimens (Fig. 2a). However, in the culture course GB cells maintained in monolayer begin to lose tumor characteristics and there is an increased number of proliferating normal cells when compared to cancer cells, while single remaining GFAP-positive cells are not undergoing mitoses (*p* < 0.005 for GB6, *p* < 0.003 for GB7, *p* < 0.011 for GB8 and *p* < 0.013 for GB9 cultures; Additional file 4: Table S4). This may be associated with their being simultaneously positive for the senescence-associated β-galactosidase (SA-β-Gal), indicating senescence occurrence (Fig. 2b).

As for the second of the tested protocols, it was initially applied by Xie et al. to establish Human Glioblastoma Cell Cutlure (HGCC) repository and it is supposed to mimic NSC-like conditions [16]. It consists of two-step establishment protocol, encompassing neurosphere formation with short-term neurosphere culture (5–7 days), followed by monolayer conditions after enzymatic disintegration of formed neurospheres. Short term maintenance of five out of ten analyzed GB cases in a form of neurospheres provided cultures of GFAP-positive cells, with only slight percentage of glioblastoma-associated stromal cells (GASCs; positive for the expression of α-SMA; Fig. 2c). The second step of this approach enabled migration and unaltered proliferation of cancer cells only for several days, however, in late passages GFAP-positive cells tended to become large, non-proliferating (statistically significant for GB6 and GB9, *p* = 0.005 and *p* = 0.045, respectively) and unable to adhere to culture plate following passage (Fig. 2d-e). Importantly, the in vivo tumor phenotype of cells was better preserved than in case of standard monolayer culture as higher number of GFAP-positive cells was observed, due to the limited proliferation of normal, GFAP-negative and α-SMA-positive cells (neurosphere culture hampers overgrowth of cancer cells by normal stromal cells in culture). As a result, the proliferation rate of normal cells in NSC-like cultures was much lower than in corresponding monolayer conditions (*p* = 0.015 for GB 6; *p* = 0.001 for GB7, *p* < 0.001 for GB8 and *p* = 0.001 for GB9; Fig. 2f-k). As mentioned, two out of ten analyzed GB specimens (GB7 and GB10) were stabilized in NSC-like conditions, and the same samples stabilized in monolayer ones as well, indicating that culturing conditions may not be as crucial in culture establishment as these are considered to be.

As we observed that the cultured cells were undergoing senescence, we decided to evaluate the percentage of SA-β-Gal positive cells in four randomly selected cultures in both analyzed conditions and different passages. Such analysis was also conducted to evaluate whether the observed phenomenon may have an impact on stabilization success. To make the analysis more profound, not only the percentage of SA-β-Gal positive cells, but also the percentage of apoptotic (using caspase 3/7 substrate; Additional file 5: Figure S5), proliferating (BrdU-positive) and other cells in these cultures was calculated (Fig. 2g-k, Additional file 4: Table S4). In case of cancer cells, the significant increase in senescent and decrease in proliferating cells during culture course was observed, irrespective to culture conditions applied (as e.g. for GB9, in monolayer with *p* = 0.032 and *p* = 0.008, for senescent and proliferating cells, respectively, and in NSC-like ones for senescent and proliferating cells with *p* = 0.028 and *p* = 0.045, respectively), while in case of normal cells, BrdU-positive cells remained on a relatively stable level in monolayer conditions (with an exception for GB9 with *p* = 0.037; Additional file 4: Table S4), while in later passages of NSC-like conditions population of normal cells was so marginal that it was not a subject of any analyses. Interestingly, despite the fact that GB7 was characterized by a relatively high percentage of senescent cells (Fig. 2h), similarly to other tumors (Fig. 2g, j-k), it finally stabilized indicating that the phenomenon of senescence itself (even observed in a high percentage of cells) may probably be not alone sufficient for stabilization failure.

### Cultured glioblastoma cells tend to undergo mitotic catastrophe that is often accompanied by senescence

**Fig. 3:**
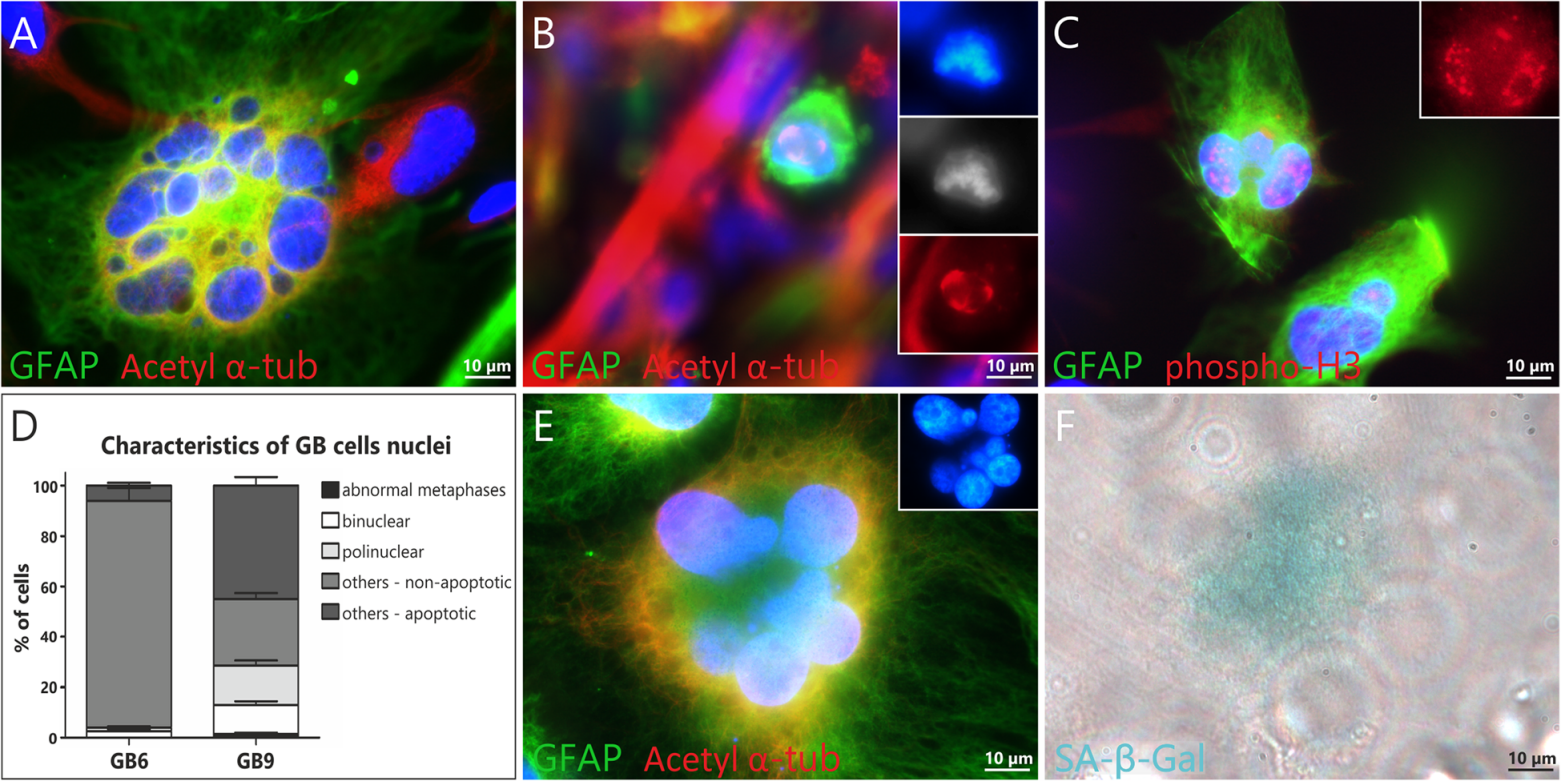
Spontaneous/idiopathic mitotic catastrophe (MC) in primary glioblastoma cultures. Polynucleated cell following MC in GB6 in early monolayer culture conditions (**a**) as well as abnormal mitoses in early passages of NSC-like conditions in GB9 are visible; (**b-c**) Multipolar spindles in GFAP positive cell abnormal mitosis (**b**) as well as polynuclear cells with asymmetric distribution of phosphorylated histone 3 may be easily detected in early passages of GB 7 in NSC-like conditions (**c**); The percentage of cells with the features of mitotic catastrophe (abnormal metaphases with spindle apparatus/chromosomal misalignments, mono- and multipolar spindles, bi- and multinuclear cells formed following MC) differs depending on case (GB6 and GB9) in the same conditions, here NSC-like ones; (**d**) Cells with the features of MC may be simultaneously SA-β-Gal positive or pass through mitotic cell death, as polynucleated senescent cell in GB9 in passage 4 of NSC-like conditions (**e-f**). The average percentage was obtained by analyzing of at least 200 cells per case (GB6 and GB9). Error bars indicate SEM

In order to get a broader insight into the mechanism underlying the inability to stabilize primary GB cultures, especially in relation to the high percentage of senescent cells, we decided to more profoundly analyze the phenomenon of mitotic catastrophe and mitotic cell death. We detected a high percentage (even up to 30%) of large, flat, bi- and multinucleated cells following mitotic catastrophe in both analyzed conditions (NSC-like and classical monolayer). Moreover, up to 5% of GFAP-positive cells undergone abnormal mitoses, with acetylated multipolar spindles emphasized by alpha-tubulin staining, as well as asymmetric distribution of phosphorylated histone H3 (Fig. 3a-d). Importantly, the majority of multinucleated cells formed as a result of mitotic catastrophe was simultaneously SA-β-Gal positive, indicating that senescence accompanies or is preceded by the mitotic catastrophe (Fig. 3e-f). One should not be surprised that the percentage of cells undergoing abnormal mitoses was marginal in comparison to cells following the mitotic catastrophe phenomenon (Fig. 3d), as the percentage of cells fixed during short-lasting metaphase cannot be high, whereas senescent cells preserve in cell culture for days or even weeks.

### Transduction with immortalizing factors does not enable to successfully stabilize GB cell culture

**Fig. 4:**
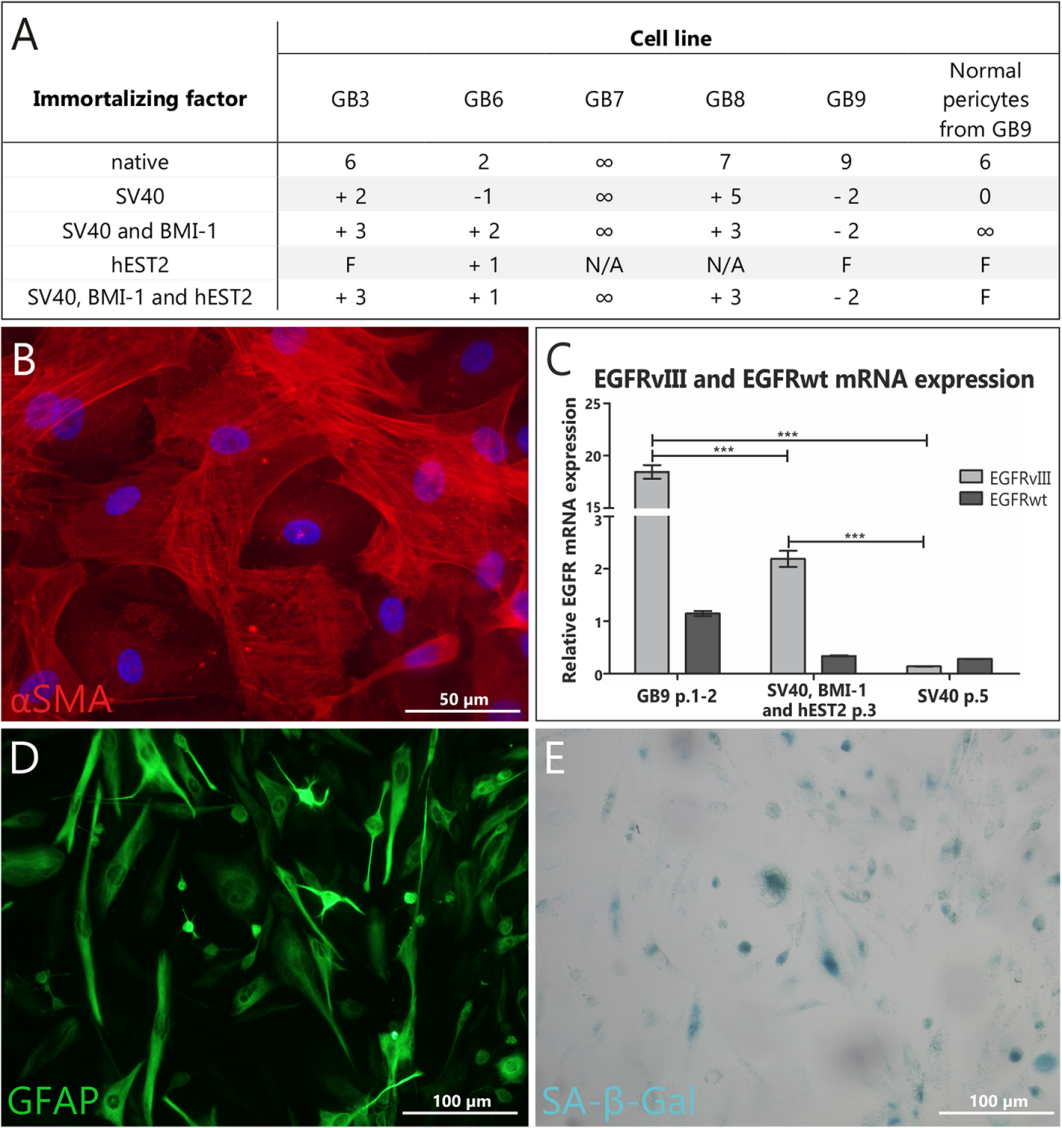
Primary glioblastoma cells transduced with immortalizing factors – none of applied factors successfully stabilized GB cell culture. **a** Table summarizing attempts to transduce GB cells at early culture stages with immortalizing factors; **b** Late passage of αSMA (+) pericytes from GB9 sample immortalized with lentivirus with *SV40 + BMI1*; **c** GB9 cells immortalized with lentivirus with *BMI + hEST2 + SV40* and *SV40* alone. Significant decrease in EGFR^vIII^ and EGFR^WT^ expression following immortalization attempt was observed; **d-e** Early passage of primary GB7 culture (mostly GFAP (+), SA-β-Gal (+) cells) treated with *SV40 + BMI*. ‘∞’ infinite proliferation; “F” cells did not get to the 1^st^ passage; ‘+‘indicates the number of passages beyond native conditions; ‘-‘ indicates the number of passages beneath native conditions; ‘0’ no effect observed; ‘NA’ not analyzed. Error bars indicate SEM. Statistical significance for Real-time PCR results was determined by two-way ANOVA analysis with post-analysis Bonferroni’s multiple comparisons tests. *, *p* < 0.05; **, *p* < 0.01; ***, *p* < 0.005

Primary cancer cells are known to be less prone to genetic manipulations than normal cells, as only few successful immortalization attempts have been described in the literature. Nevertheless, we decided to transduce five out of ten analyzed glioblastoma specimens with selected immortalizing factors (Fig. 4a). None of the factors applied, either alone or in combination, enabled to stabilize GB culture, as GB7 cells stabilized even in monolayer culture and NSC-like conditions, without the need of any immortalization attempts. In contrast, normal human pericytes from GB9 specimen were successfully immortalized using combination of *SV40* and *BMI-1* factors (Fig. 4a-b). Obtained results indicate that transduction with immortalization factors generally may lead to increase in the number of GB cells passages, but is not sufficient to maintain tumor-characteristic molecular changes (significant decrease in *EGFR*^*WT*^ and *EGFR*^*vIII*^ expression was observed following immortalization attempt; Fig. 4c), or to avoid senescence (Fig. 4d-e).

### Sequential in vitro*/*in vivo cultivation maintains original genotype and phenotype of glioblastoma cells for the longest period

**Fig. 5:**
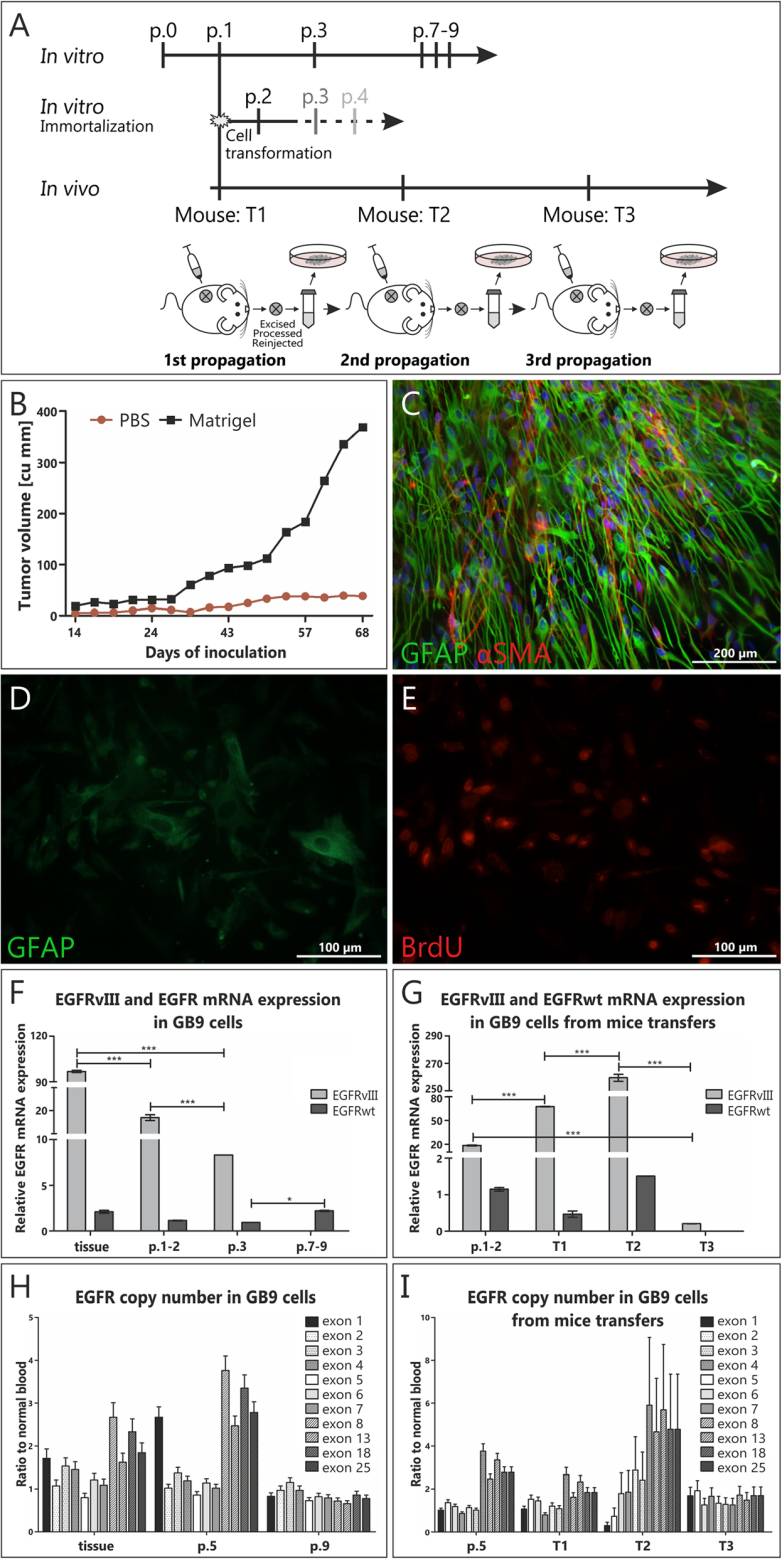
Co-injecting glioblastoma tumor cells with basement membrane matrix proteins enables their growth in vivo. **a** Schematic workflow of sequential in vitro*/*in vivo culturing of glioblastoma cells. Primary GB tumors from three patients (GB6, GB8 and GB9) were maintained for total ± 30 weeks in vivo vs ± 8 weeks in vitro, before they lost original mutational profile. *P – passage, T – propagation* in vivo; **b** Two separate groups were injected with <10^4^ cells/mouse from fresh GB9 spheres, either suspended in PBS or Matrigel; **c** Strong GFAP (+) cells from early GB9 sphere were injected subcutaneously into mice; **d-e** BrdU (+) GB9 cells with decreased GFAP expression and altered morphology removed from mice after 3^rd^ transfer; **f-g** EGFR^vIII^ and EGFR^WT^ expression in GB9 cells from mice transfers and further in vitro propagations; **h-i** *EGFR*^*vIII*^ (characterized by deletion of exons 2–7) copy number gain in passage 0 and early transfer in mice is gradually lost both, in further passages of in vitro culture or mice transfers. For MLPA results error bars indicate SD, while for Real-time PCR results SEM. Statistical significance calculated by two-way ANOVA analysis with post-analysis Bonferroni’s multiple comparisons test. *, *p* < 0.05; **, *p* < 0.01; ***, *p* < 0.005

To test the capacity of in vitro processed GB cells to initiate tumor formation, we carried out subcutaneous transplantation into immunocompromised mice. Three out of ten GB specimens were analyzed in this approach (GB6, 8 and 9) and each time tumor tissue was extracted, minced just like tissue specimen, and either re-injected the same way into another mice (next transfer) or cultured in vitro (Fig. 5a). Preliminary in vivo analyses indicated that for efficient tumor growth cells need to be mixed with Matrigel prior to injection (Fig. 5b). Importantly, injected cells were analyzed in terms of phenotypic (Fig. 5c-g) and genotypic (Fig. 5f-i) tumor characteristics in each step of the procedure. Following 2^nd^ (in case of GB8) or 3^rd^ resection (in case of GB6 and GB9) not only the original mutational profile of analyzed cells was lost, as monitored by molecular analyses (mostly MLPA and Real-time PCR; Fig. 5h-i), but also the decrease in tumor-characteristic phenotype (loss of GFAP expression; Fig. 5d) was observed with the fraction of remaining proliferating cells (BrdU-positive; Fig. 5e). Therefore, such conditions enabled to maintain up to 3 subsequent transfers of GB cells in mice, while maintaining their original genotype and phenotype as well as their proliferation capacity. This approach can be basically considered an “*in vivo* incubator”, aimed to propagate primary cells.

### Idiopathic senescence and apoptosis tend to occur not only in glioblastoma primary cultures, but also in stable cell lines

**Fig. 6:**
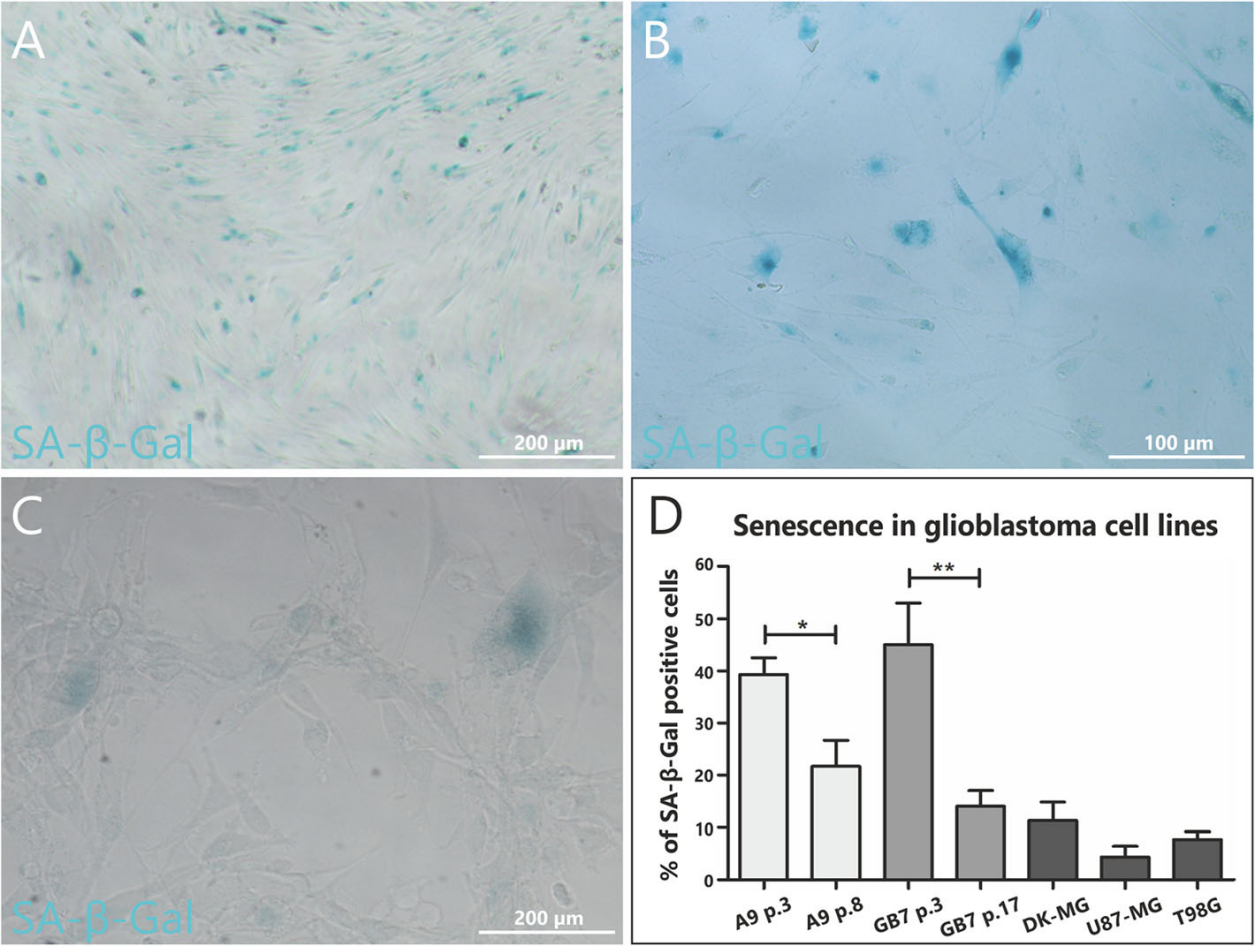
Stabilized glioblastoma cell lines in standard 10% serum monolayer conditions are characterized by a fraction of SA-β-Gal (+) cells. Comparison of A9 cell line in early 3^rd^ (**a**) and late 8^th^ passage (**b**). Representative picture showing senescent cells in commercially available cell line U87-MG (**c**). Comparison of spontaneous in vitro senescence in primary glioblastoma cultures stabilized in our department (A9, GB10) and commercially available GB stable cancer cell lines (**d**). The average percentage was obtained by analyzing of at least 200 cells *per* case from indicated passages (A9, GB7) or for stable GB lines (DK-MG, U87-MG and T98G). The number of SA-β-Gal (+) cells statistically differs for A9 line between passage 3 and passage 8 as well as for GB7 line between passage 3 and passage 17 (*p* < 0.05). Error bars indicate SEM. Statistical significance calculated by paired Student’s t-test. *, *p* < 0.05; **, *p* < 0.01; ***, *p* < 0.005

Considering senescence not only as a cause of inability to stabilize glioblastoma cell cultures but generally as an inherent feature of some cell subpopulations in tumor mass, it is important to analyze this phenomenon in stable cell lines. The most frequently applied senescence marker – SA-β-Gal activity, enabled to confirm the presence of this phenomenon in most common stable GB cell lines, as well as glioblastoma cultures previously stabilized in our department (Fig. 6). Idiopathic senescence (occurring without any external stimuli) was detected in small populations (approx. 10%) of U87-MG, T98G, DK-MG lines and, interestingly, in A9 (previously stabilized by the authors) and GB7 (analyzed and successfully stabilized in this article) cultures (Fig. 6a-d). Moreover, this phenomenon was detected not only in serum free or low serum conditions but also in fully conditioned media, even as soon as 24 h after new passage. Finally, it is worth to emphasize that despite the fact that A9 cell line as well as GB7 were characterized with high percentage of SA-β-Gal positive cells in early passages, these were finally stabilized (Fig. 6a-b), suggesting that senescence may not constitute the sole culprit for stabilization failure.

## Discussion

Despite many years of extensive research glioblastoma, the most malignant brain tumor, remains incurable. This is not only associated with its location, severly limiting treatment options, but also high intratumoral heterogeneity [3, 4, 12, 16]. There are very few established, indefinitely proliferating GB cell lines, characterized by molecular alterations that are commonly found in vivo [4]. Therefore, there is a need to develop the reliable GB in vitro testing platform. Unfortunately, establishment of primary glioblastoma cultures, definately more heterogenous and better reflecting in vivo tumor state, is difficult.

In theory, cancer progression model, based on transition from preneoplastic to neoplastic phase, requires cancer cells bypassing cellular senescence and apoptosis [1]. However, despite being one of the most aggressive tumors, GB cells tend to undergo idiopathic senescence and apoptosis, in some cases even quite extensive, in early passages during in vitro culturing [4]. In very few cases of established GB cultures, notably these characterized by a specific combinations of molecular alterations that are almost never found in vivo, these phenomena are counterbalanced by proliferation enabling culture establishment [4]. It remains unknown what determines stabilization success and whether is it associated with culture conditions, glioblastoma subtype or molecular characteristics of the tumor. In this paper, cells that failed stabilization attempts (8 out of 10 analyzed cases) were cultured not only according to NSC conditions-mimicking protocol (previously applied by Xie et al.), but also in standard monolayer culture conditions. This clearly indicates that optimization of culture conditions does not constitute the ultimate solution to stabilization problem of primary cancer cells. We have previously detected the atypical combination of *TP53* and *CDKN2A* mutations in almost half cases of stabilized GB cultures [4]. As these alterations are considered mutually exclusive in glioblastoma, it may suggest that they predispose cells to stabilization. On the other hand, their very rare in vivo occurrence indicates that when stabilized, primary GB cells tend to deviate from the original genotype. Indeed, during in vitro maintenance uncontrolled selection of cells with specific mutation or phenotype can be observed [3, 12, 16] and due to glioblastoma genomic instability additional genetic alterations may commonly occur, especially late in the culture course. One of the cultures stabilized in the current study (GB7) was initially characterized by *TP53* mutation, while in both established cultures (GB7 and GB10) we detected deletion of one allele of *PTEN* further in the culture course. Therefore, successful stabilization of these particular cases might have been associated with positive selection of cells characterized by the deletion of these two suppressor genes. Basing on the obtained results we cannot directly confirm our previous assumptions regarding the co-existence of tumor suppressor mutations. Still, it seems that lack of cell cycle control may facilitate cancer cells transfer and maintenance in vitro. Moreover, we have previously described that there is a higher frequency of homozygous mutations in tumor suppressors as well as mutations resulting in lack of tumor suppressor proteins in cell lines when compared to surgical samples. Hence, there seems to be a complete loss of function of tumor suppressor genes in time [23]. On the other hand, mesenchymal GB subtype is suggested to be more prone to stabilization, even if patient was diagnosed with another GB subtype [16], what seems to be reasonable, as cells in monolayer conditions attach to the culture vessel and compete with normal stromal cells [24]. Indeed, some researchers even observed the transition from mesenchymal type (observed in vitro) to other phenotypes in xenografts [16]. In general, all these data clearly indicate discrepancies between in vivo and in vitro analytical models what can be of utmost importance in terms of drugs testing perspective.

Another important aspect for cancer cell culturing is the lack of unified approach for primary cells establishment and conditions for their further maintenance. Currently, over 20 different protocols are in use [14], hence obtained results cannot constitute a subject of reliable comparison. Indeed, there are a lot of discrepancies between laboratories working in the field of GB primary cultures. Research group headed by Pollard [12] indicated inconsistencies between NSC-like adherent conditions and neurosphere 3D cultures, what is in line with our results and those obtained by other research teams [4, 12, 16, 25, 26]. This report was immediately criticized by Reynolds and Vescovi [13] as this group claimed that their processing of 150 GB specimens resulted in 100% efficiency in cell culture establishment using neurosphere-based approach. Intriguingly, this research group claimed that they were able to passage these cells approximately 50 times a year (up to 150 passages), which can be considered quite an achievement. Still, it is very hard to comprehend, since neither our team nor any other laboratory reported complete efficiency of primary GB cells stabilization, especially when neurospheres formation was involved. Usually, establishment rate is estimated to be 33–47%, while the number of passages reached by cultures in NSC-like conditions do not generally exceed 10 (when proper molecular analyses during the culture course are included) [12, 16]. Actually, it was demonstrated that the subtype of GB cells matched tumor of origin only in 45% of cases, mostly due to high in vivo tumor heterogeneity [16]. These examples clearly indicate that results of primary GB cells establishment and analyses conducted using such models should be analyzed more critically.

As mentioned, discrepancies concerning culture establishment may result from the fact that the term ‘stable cell line’ is not uniformly defined. In our laboratory, we consider cancer cell line stabilized when it reaches at least 50 passages (approx. twice as many as normal cells do) and shows any molecular changes at DNA level. The percentage of cultures that stabilize in our laboratory has been constant for many years, despite the application of new culturing approaches, culture media, supplements or coatings and is not directly associated with the technical abilities of the person performing the procedure. It seems that not all researchers tend to follow such strict criteria, as the percentage of stabilized cell lines reported in the literature is often overestimated. Cell line cannot be considered stable when it reached only several passages (e.g. only up to 10) or molecular status of these allegedly stabilized cells is not verified [24]. Unfortunately, established cultures are often not molecularly analyzed at various time points. Nevertheless, it can be considered quite encouraging that articles analyzing the aspect of primary cells culturing from a more complex point of view begin to be published [27]. One can criticize that stabilization success rate in this study is less than average, however, strict criteria used in our laboratory may ensure reliability of results.

Since appearance of senescence and apoptosis in primary neoplastic cells is considered spontaneous (idiopathic), recognizing and describing the mechanism of these phenomena, which tend to occur at early passages of GB culture, remain crucial to circumvent culturing difficulties. We are aware of the fact that senescence and apoptosis, which can be very extensive in some cases, are somehow associated with stabilization failure and may constitute a result of mitotic catastrophe. It is not the first report associating senescence-like phenotype (SLP) with mitotic catastrophe. Indeed, Eom et al. pointed out that cells may firstly undergo mitotic catastrophe and then enter a temporary senescence-like arrest prior to cell death [28]. Moreover, Wang et al. demonstrated that GB cells with normal TP53 protein tend to exhibit SLP in response to inhibition of topoisomerase I, while cells with mutated TP53 undergo apoptosis [29]. On the other hand, TP53 inhibition in fibrosarcoma cells was found to be associated with decreased drug-induced SLP and increased mitotic catastrophe [30]. Although in mentioned studies these phenomena were not spontaneous, in this article we made an attempt not only to test various methods of GB culture establishment, but, most importantly, to try to provide a greater insight into the mechanisms underlying inability to stabilize these cells in vitro. Therefore, lentiviral vectors and in vitro*/*in vivo cultivation approaches were mostly applied in the article not as a method of culture establishment, but rather as an attempt to understand the causes of the difficulties in stabilization of primary cancer cells.

Based on the approaches that were actually developed for normal cells, we put forward the hypothesis that in vitro immortalization may enable longer propagation of primary GB cells. Several immortalizing factors were selected considering genetic pathways involved in cancer cells senescence. Transduction of primary cell cultures with BMI-1 (inactivating CDKN2A/RB pathway), SV40 (binding and inactivating tumor suppressor protein TP53) and hEST2 (generally up-regulated in tumor cells and during immortalization, preventing senescence resulting from telomere shortening) was insufficient to provide infinite proliferation. Since it is well established that both pathways, TP53/p14 and p16, are essential to induce replicative senescence [31] we assume that this mechanism is not associated with the observed phenomena. Neoplastic cells are thought to maintain high proliferation capacity mainly due to their telomerase activity – large ribonucleoprotein complex (dependent on hEST2), regulating cell replicative lifespan through stabilization and extension of telomeres [32]. Repression of telomerase or lack of its activity finally leads to cellular senescence, apoptosis or premature aging [33]. Various reports have demonstrated link between neoplastic progression and telomerase activity emphasizing the fact that only minority (10–15%) of malignant cells does not possess upregulated hEST2 expression [34]. However, recently it was proven that elongation of telomeres does not eliminate occurrence of senescence [35]. Moreover, inactivation of proteins involved in cell cycle control is possibly not enough to circumvent senescence, despite the fact that this was suggested by the results of our previous analyses, indicating that proper balance between senescent/apoptotic and proliferating cells may be achieved only when specific genetic alterations ensuring lack of cell cycle control (e.g. a combination of *TP53* mutation and *CDKN2A* homozygous deletion) co-exist in the cell [4].

Considering the fact that all analyzed in vitro conditions might have an impact on triggering senescence and apoptosis, we decided to test sequential in vitro*/*in vivo model of culturing glioblastoma-derived cells, as it was reported that the majority of glioblastoma subtypes can efficiently give rise to tumors when implanted in vivo, with two thirds retaining characteristics of original primary tumor tissue [16]. Despite the fact that this approach did not enable to stabilize analyzed GB cases, so far it seems to be the most promising option for long-term culturing of these cells. Above all, it facilitates fast propagation of native, unaltered cancer cells for the purpose of e.g. screening of molecules with antineoplastic potential in both, in vivo and in vitro conditions. It is especially important as in the majority of cases the amount of material directly derived from patients is not sufficient for standardized analyses and further statistical assessment. Importantly, despite the fact that our model was based on subcutaneous GB cells injection, it still enabled to impede growth arrest and phenotypic changes of tumor cells longer than all tested in vitro approaches.

One of the most important questions is whether the inability to evade senescence/apoptosis by the majority of primary GB cultures observed in the present study, is a feature inherent in the behavior of these cells. Despite our many years of research on this topic, we are still unable to unequivocally determine whether senescence constitutes the major reason of stabilization failure or is it a feature characteristic to GB cells. It has to be mentioned that senescent glioblastoma cells have been reported to be present in vivo [36], however, as we demonstrated in this paper, the percentage of senescent cells tends to increase with in vitro passages. To make it even more complicated, our team and others demonstrated that a small, but rather constant, population of senescent, and even apoptotic cells is also present in stable cancer cell lines [6, 37, 38]. Hence, we decided to evaluate the percentage of senescent cells in several stable glioblastoma cell lines. Our analyses indicated a considerable diversity both, in the presence and percentage of senescent cells – cells positive for SA-β-Gal activity were detected within T98G, U87-MG and DK-MG lines, with the last line characterized by the highest percentage of senescent cells. These results may support the theory of dual – anti- and pro-neoplastic role of senescence [39], complicating manipulations of this phenomenon in anticancer research as well as unequivocal understanding of the mechanism of this process and its implications. Finally, not only idiopathic senescence, but also idiopathic/spontaneous apoptosis was observed in primary and stabilized GB cell lines. These data suggest that tumor is highly heterogeneous not only from a molecular, but also a functional point of view, meaning that not all cancer cells are immortal, at least under in vitro conditions. Such intratumoral heterogeneity is associated with the fact that not single cells, but rather cell subpopulations tend to exhibit specific functions (e.g. with part of cells being immortal, while another part being associated with secretory/senescent phenotype). Therefore, not only cancer stem cells should be considered in such a context, as these interdependencies may be more complicated, as demonstrated on an example of stable cells lines (“fixed” percentage of senescent/apoptotic cells).

## Conclusions

The major focus of this study was not only an attempt to compare culturing methods with the occurrence of phenomena impeding in vitro stabilization of GB cells, but also an attempt to test the impact of environment (in vivo analyses) or molecular aspects (immortalization approach). Overall, we aimed to associate establishment inhibitory phenomena with some possible underlying mechanisms. In the majority of primary glioma cultures, there has to be an imbalance towards apoptosis and senescence, following few weeks of rapid proliferation. Our results indicate that these GB cell lines that were successfully established were both stabilized irrespective of culture conditions and without a strict dependence on proteins controlling cell cycle. Nonetheless, the mechanism underlying the imbalance between proliferation and other observed phenomena remains elusive. Most importantly, its determination could have significant implications for design of novel anticancer therapies. Our data clearly indicate that senescence cannot be inhibited using several cell biology or genetic engineering approaches.

## Supplementary information files

**Additional file 1: Table S1.** Sequences of primers used to prepare lentiviral vectors with immortalizing factors. attB flanking sites were applied for further usage in Gateway system. (DOCX 14 kb)

**Additional file 2: Table S2.** Antibodies used in immunofluorescence analyses. (DOCX 15 kb)

**Additional file 3: Table S3.** Molecular changes during passages in each analyzed glioblastoma cell culture. Sanger sequencing for *IDH1* codon 132 and *TP53* exons 4–8; wt – wild type; amp – amplification; MLPA ratio was interpreted as normal copy number (0.7–1.3); deletion of one allele (0.35–0.65), deletion of both alleles (0), gain of one allele (e.g. trisomy) (1.35–1.55), gain of more than one allele (1.6–2.2), while the value in range of 0.1–0.3 was considered to result from the analysis of heterogeneous materialin which deletion was detected; ±SD. Results of Real-time PCR for *EGFR*^*WT*^ and *EGFR*^*vIII*^ expression were analyzed as previously described [23]. Results of Real-time PCR for DNA copy number were analyzed as described in ‘Materials & Methods’ section. (DOCX 63 kb)

**Additional file 4: Table S4.** The results of paired Student’s t-test for the comparison of cell biology features of neoplastic and normal cells in glioblastoma primary cultures in different conditions. (DOCX 17 kb)

**Additional file 5: Figure S5.** Apoptosis of glioblastoma cells. Representative images showing classical apoptotic nuclei with TP53 accumulation (A) as well as activity of the synthetic Caspase 3/7 reporter in early passages of GB9. The number of Caspase 3/7 positive cells was higher in NSC-like conditions (C) than in monolayer (B) (quantitative data are shown in Fig. 3g-k and Additional file 4: Table S4). (DOCX 713 kb)

## Data Availability

All data generated or analyzed during this study are included in this published article and its supplementary information files.

## Abbreviations

AGM: Astrocyte Growth Medium
BrdU: Bromodeoxyuridine
FISH: Fluorescent in situ hybridization
GB: Glioblastoma
HBSS: Hank’s Balanced Salt Solution
MLPA: Multiplex ligation-dependent probe amplification
NBM: Neurobasal medium
NSC: Neural stem cell
PEI: Polyethylenimine
PFA: Paraformaldehyde
SA: Senescence-associated
