# Supplementary material for: A way to understand idiopathic senescence and apoptosis in primary glioblastoma cells – possible approaches to circumvent these phenomena
Source: BMC Cancer. 2019 Sep 14;19:923. doi: 10.1186/s12885-019-6130-2 (PMC6744717; doi:10.1186/s12885-019-6130-2)
Supplement: Supplementary file 1 — Additional file 1: Table S1. Sequences of primers used to prepare lentiviral vectors with immortalizing factors. attB flanking sites were applied for further usage in Gateway system. (DOCX 14 kb) [file 12885_2019_6130_MOESM1_ESM.docx]

**Table S1.** Sequences of primers used to prepare lentiviral vectors with immortalizing factors. *att*B flanking sites were applied for further usage in Gateway system.

| **Primer** | **Gene-specific nucleotides** | **Cell line** |
| --- | --- | --- |
| hEST2 *att*B1 | ATGCCGCGCGCTCCCCGCTGCCGAGCCGTG | NTERA-2 |
| hEST2 *att*B2 | GTCCAGGATGGTCTTGAAGTCTGAGGGCAG |  |
| BMI-1 *att*B1 | ATGCATCGAACAACGAGAATCAAGATC | HeLa |
| BMI-1 *att*B2 | ACCAGAAGAAGTTGCTGATGACCCATT |  |
| SV40 *att*B1 | ATGGATAAAGTTTTAAACAGAGAGGAATCTTTGCAGC | HEK293T |
| SV40 *att*B2 | TGTTTCAGGTTCAGGGGGAG |  |
