# Supplementary material for: A way to understand idiopathic senescence and apoptosis in primary glioblastoma cells – possible approaches to circumvent these phenomena
Source: BMC Cancer. 2019 Sep 14;19:923. doi: 10.1186/s12885-019-6130-2 (PMC6744717; doi:10.1186/s12885-019-6130-2)
Supplement: Supplementary file 2 — Additional file 2: Table S2. Antibodies used in immunofluorescence analyses. (DOCX 15 kb) [file 12885_2019_6130_MOESM2_ESM.docx]

**Table S2.** Antibodies used in immunofluoresence analyses.

| Antibody | Host | Manufacturer | Dilution |
| --- | --- | --- | --- |
| BrdU | Mouse | Sigma-Aldrich (B 8434) | 1: 500 |
| GFAP | Rabbit | Abcam (ab7260) | 1:1000 |
| GFAP | Mouse | Millipore (mab360) | 1:400 |
| α-SMA | Mouse | R&D Systems (mab1420) | 1:400 |
| acetylated α Tubulin | Mouse | Santa Cruz Biotechnology (sc-23950) | 1:500 |
| phospho-Histone H3 | Mouse | Santa Cruz Biotechnology (sc-374669) | 1:50 |
| anti-mouse Alexa Fluor 594 | Donkey | Life Technologies | 1:500 |
| anti-rabbit Alexa  Fluor 488 | Donkey | Life Technologies | 1:500 |
