# Supplementary material for: A way to understand idiopathic senescence and apoptosis in primary glioblastoma cells – possible approaches to circumvent these phenomena
Source: BMC Cancer. 2019 Sep 14;19:923. doi: 10.1186/s12885-019-6130-2 (PMC6744717; doi:10.1186/s12885-019-6130-2)
Supplement: Supplementary file 3 — Additional file 3: Table S3. Molecular changes during passages in each analyzed glioblastoma cell culture. Sanger sequencing for IDH1 codon 132 and TP53 exons 4–8; wt – wild type; amp – amplification; MLPA ratio was interpreted as normal copy number (0.7–1.3); deletion of one allele (0.35–0.65), deletion of both alleles (0), gain of one allele (e.g. trisomy) (1.35–1.55), gain of more than one allele (1.6–2.2), while the value in range of 0.1–0.3 was considered to result from the analysis of heterogeneous materialin which deletion was detected; ±SD. Results of Real-time PCR for EGFRWT and EGFRvIII expression were analyzed as previously described [23]. Results of Real-time PCR for DNA copy number were analyzed as described in ‘Materials & Methods’ section. (DOCX 63 kb) [file 12885_2019_6130_MOESM3_ESM.docx]

**Table S3.** Molecular changes during passages in each analyzed glioblastoma cell culture. Sanger sequencing for IDH1 codon 132 and TP53 exons 4-8; wt – wild type; amp – amplification; MLPA ratio was interpreted as normal copy number (0.7-1.3); deletion of one allele (0.35-0.65), deletion of both alleles (0), gain of one allele (e.g. trisomy) (1.35-1.55), gain of more than one allele (1.6-2.2), while the value in range of 0.1-0.3 was considered to result from the analysis of heterogeneous material in which deletion was detected; ± SD. Results of Real-time PCR for EGFR^WT^ and EGFR^vIII^ expression were analyzed as previously described [23]. Results of Real-time PCR for DNA copy number were analyzed as described in ‘Materials & Methods’ section.

|  |  |  |  | **Frozen sample** | **Monolayer** | | | **NSC-like** | |
| --- | --- | --- | --- | --- | --- | --- | --- | --- | --- |
| **GB1** | **Source** | **Analysis** | |  | **p. 1** | **p. 9** | **-** | **-** | **-** |
|  | mRNA | RT Real-time  PCR | EGFRwt expression | 7.91±0.05 | **-** | 5.85±0.63 | **-** | **-** | **-** |
|  |  |  | EGFRvIII expression | 0.23±0.05 | **-** | 0.17±0.06 | **-** | **-** | **-** |
|  | DNA | Real-time  PCR | Total EGFR | 75.1±8.11 | **-** | 15.91±3.99 | **-** | **-** | **-** |
|  |  |  | EGFRwt | 2.51±0.66 | **-** | 2.91±0.81 | **-** | **-** | **-** |
|  | DNA | MLPA | EGFRwt | - | **-** | 1.4±0.04 | **-** | **-** | **-** |
|  |  |  | EGFRvIII | 1.59±0.43 | **-** | 1.29±0.04 | **-** | **-** | **-** |
|  |  |  | CDKN2a | 0.58±0.06 | **-** | 0.98±0.03 | **-** | **-** | **-** |
|  |  |  | PTEN | 0.85±0.09 | **-** | 0.96±0.03 | **-** | **-** | **-** |
|  |  |  | other changes | MDM4 (1.79±0.49); KIT (1.9±0.52);  AMER (0.45±0.5);  CDKN2B (0.3±0.03); MET (2.18±0.6);  KDR (1.55±0.42); PDGFRA (2.07±0.4); RET (0.51±0.14) | **-** | not detected | **-** | **-** | **-** |
|  | DNA | Sanger sequencing/IHC | IDH1 status | wt | **-** | - | **-** | **-** | **-** |
|  |  |  | TP53 status | wt | **-** | - | **-** | **-** | **-** |
|  | cells | FISH | EGFR amp/ polysomy of chromosome 7 | - | amp | - | **-** | **-** | **-** |
| **GB2** | **Source** | **Analysis** | |  | **p. 1** | **p. 9** | **-** | **-** | **-** |
|  | mRNA | RT Real-time  PCR | EGFRwt expression | 12.45±1.14 | **-** | 4.29±0.26 | **-** | **-** | **-** |
|  |  |  | EGFRvIII expression | 0.014±0.006 | **-** | 0±0 | **-** | **-** | **-** |
|  | DNA | Real-time  PCR | Total EGFR | 1.93±0.19 | **-** | 2.33±0.96 | **-** | **-** | **-** |
|  |  |  | EGFRwt | 2.41±0.43 | **-** | 1.74±0.48 | **-** | **-** | **-** |
|  | DNA | MLPA | EGFRwt |  | **-** | 1.16±0.03 | **-** | **-** | **-** |
|  |  |  | EGFRvIII | 2.42±0.33 | **-** | 1.17±0.03 | **-** | **-** | **-** |
|  |  |  | CDKN2a | 0.62±0.06 | **-** | 1.00±0.03 | **-** | **-** | **-** |
|  |  |  | PTEN | 0.64±0.06 | **-** | 1.01±0.03 | **-** | **-** | **-** |
|  |  |  | other changes | MDM2 (4.67±0.64);  MET (3.88±0.53);  AURKA (2.07±0.28);  BRAF (1.66±0.23);  RET (0.5±0.07) | **-** | not detected | **-** | **-** | **-** |
|  | DNA | Sanger sequencing/IHC | IDH1 status | - | **-** | - | **-** | **-** | **-** |
|  |  |  | TP53 status | - | **-** | - | **-** | **-** | **-** |
|  | cells | FISH | EGFR amp/polysomy of chromosome 7 | - | single cells with amp | - | **-** | **-** | **-** |
| **GB3** | **Source** | **Analysis** | |  | **p. 1** | **p. 3** | **p. 4** | **-** | **-** |
|  | mRNA | RT Real-time  PCR | EGFRwt expression | 2.61±0.86 | 2.85±0.13 | 1.86±0.34 | 1.72±0.16 | **-** | **-** |
|  |  |  | EGFRvIII expression | 21.61±3.76 | 9.17±3.79 | 3.26±0.58 | 4.52±0.85 | **-** | **-** |
|  | DNA | Real-time  PCR | Total EGFR | 215.7±38.41 | 78.24±16.44 | 11.69±6.96 | 15.42±4.61 | **-** | **-** |
|  |  |  | EGFRwt | 1.91±0.29 | 1.89±0.4 | 1.69±0.23 | 2.29±0.76 | **-** | **-** |
|  | DNA | MLPA | EGFRwt | - | 4.89±0.63 | 1.42±0.04 | 0.95±0.04 | **-** | **-** |
|  |  |  | EGFRvIII | 1.54±0.21 | 34.89±4.52 | 4.44±0.11 | 1.04±0.04 | **-** | **-** |
|  |  |  | CDKN2a | 0.37±0.05 | 0 | 1.00±0.03 | 0.87±0.03 | **-** | **-** |
|  |  |  | PTEN | 2.37±0.48 | 0.56±0.07 | 0.98±0.02 | 0.94±0.04 | **-** | **-** |
|  |  |  | other changes | MET (1.69±0.23) | not detected | not detected | not detected | **-** | **-** |
|  | DNA | Sanger sequencing/IHC | IDH1 status | p.R132H | - | - | - | **-** | **-** |
|  |  |  | TP53 status | p.P190L | - | - | - | **-** | **-** |
|  | cells | FISH | EGFR amp/polysomy of chromosome 7 | - | amp | - | - | **-** | **-** |
| **GB4** | **Source** | **Analysis** | |  | **p. 1** | **p. 4** | **-** | **-** | **-** |
|  | mRNA | RT Real-time  PCR | EGFRwt expression | 0.95±0.14 | 0.85±0.28 | 1.37±0.67 | **-** | **-** | **-** |
|  |  |  | EGFRvIII expression | 0 | 0 | 0 | **-** | **-** | **-** |
|  | DNA | Real-time  PCR | Total EGFR | 1.65±0.32 | 1.98±0.56 | 2.07±0.36 | **-** | **-** | **-** |
|  |  |  | EGFRwt | 2.11±0.39 | 2.63±0.73 | 2.63±0.73 | **-** | **-** | **-** |
|  | DNA | MLPA | EGFRwt | 0.92±0.11 | 0.99±0.06 | 0.92±0.11 | **-** | **-** | **-** |
|  |  |  | EGFRvIII | 0.72±0.09 | 0.98±0.06 | 0.65±0.08 | **-** | **-** | **-** |
|  |  |  | CDKN2a | 0.09±0.01 | 0.31±0.02 | 0.09±0.01 | **-** | **-** | **-** |
|  |  |  | PTEN | 1.04±0.18 | 1.01±0.06 | 1.04±0.14 | **-** | **-** | **-** |
|  |  |  | other changes | CCND1 (1.41±0.17);  AURKA (1.74±0.21);  BRAF (1.53±0.19) | not detected | not detected | **-** | **-** | **-** |
|  | DNA | Sanger sequencing/IHC | IDH1 status | p.R132H | - | - | **-** | **-** | **-** |
|  |  |  | TP53 status | wt | - | - | **-** | **-** | **-** |
|  | cells | FISH | EGFR amp/polysomy of chromosome 7 | - | not detected | - | **-** | **-** | **-** |
| **GB5** | **Source** | **Analysis** | |  | **p. 1** | **p. 3** | **p. 4** | **-** | **-** |
|  | mRNA | RT Real-time  PCR | EGFRwt expression | 0.25±0.14 | 0.81±0.07 | 1.23±0.04 | 1.52±0.26 | **-** | **-** |
|  |  |  | EGFRvIII expression | 0 | 0 | 0 | 0 | **-** | **-** |
|  | DNA | Real-time  PCR | Total EGFR | 1.96±0.27 | 2.15±0.69 | 1.45±0.89 | 2.33±1.01 | **-** | **-** |
|  |  |  | EGFRwt | 1.74±0.36 | 1.76±0.46 | 2.91±1.21 | 1.99±0.47 | **-** | **-** |
|  | DNA | MLPA | EGFRwt | 1.79±0.1 | 1.47±0.07 | 0.92±0.11 | 1.08±0.03 | **-** | **-** |
|  |  |  | EGFRvIII | 0.49±0.03 | 1.37±0.06 | 0.65±0.08 | 1.07±0.03 | **-** | **-** |
|  |  |  | CDKN2a | 0 | 0 | 0.09±0.01 | 1.03±0.03 | **-** | **-** |
|  |  |  | PTEN | 1.11±0.06 | 0.62±0.03 | 1.04±0.13 | 0.96±0.03 | **-** | **-** |
|  |  |  | other changes | PDGFRA (19.3±1.1);  MDM2 (0.28±0.02); NFKB1A (1.94±0.11);  CDK4 (6.74±0.38);  KIT (2.36±0.42);  KDR (2.03±0.36); CDKN2B (0.1±0.01) | CDK4 (4.77±0.22);  PDGFRA (20.19±1.3) | not detected | not detected | **-** | **-** |
|  | DNA | Sanger sequencing/IHC | IDH1 status | wt | - | - | - | **-** | **-** |
|  |  |  | TP53 status | wt | - | - | - | **-** | **-** |
|  | cells | FISH | EGFR amp/polysomy of chromosome 7 | - | not detected | - | - | **-** | **-** |
| **GB6** | **Source** | **Analysis** | |  | **p. 2** | **-** | **-** | **p. 2** | **-** |
|  | mRNA | RT Real-time  PCR | EGFRwt expression | 0.88±0.35 | 1.17±0.29 | **-** | **-** | 1.37±0.14 | **-** |
|  |  |  | EGFRvIII expression | 0 | 0 | **-** | **-** | 0 | **-** |
|  | DNA | Real-time  PCR | Total EGFR | 2.03±0.06 | 2.79±1.42 | **-** | **-** | 2.75±1.66 | **-** |
|  |  |  | EGFRwt | 1.84±0.74 | 2.86±1.19 | **-** | **-** | 3.01±1.5 | **-** |
|  | DNA | MLPA | EGFRwt | 1.32±0.13 | 1.09±0.08 | **-** | **-** | 1.00±0.06 | **-** |
|  |  |  | EGFRvIII | 0.35±0.04 | 0.98±0.07 | **-** | **-** | 1.12±0.07 | **-** |
|  |  |  | CDKN2a | - | 0.97±0.06 | **-** | **-** | 0.90±0.06 | **-** |
|  |  |  | PTEN | 2.08±0.21 | 1.02±0.07 | **-** | **-** | 0.93±0.06 | **-** |
|  |  |  | other changes | MDM2 (0.3±0.03);  NFKB1A (1.85±0.18) | TP53 (0.55±0.04) | **-** | **-** | TP53 (0.6±0.04) | **-** |
|  | DNA | Sanger sequencing/IHC | IDH1 status | wt | - | **-** | **-** | - | **-** |
|  |  |  | TP53 status | p.P190L | - | **-** | **-** | - | **-** |
|  | cells | FISH | EGFR amp/polysomy of chromosome 7 | - | not detected | **-** | **-** |  | **-** |
| **GB7**  **stabilized** | **Source** | **Analysis** | |  | **p. 1** | **p. 5** | **-** | **-** | **-** |
|  | mRNA | RT Real-time  PCR | EGFRwt expression | 0.41±0.07 | 0.34±0.1 | 0.66±0.11 | **-** | **-** | **-** |
|  |  |  | EGFRvIII expression | 0 | 0 | 0 | **-** | **-** | **-** |
|  | DNA | Real-time  PCR | Total EGFR | 3.25±1.89 | 2.75±1.75 | 1.69±0.63 | **-** | **-** | **-** |
|  |  |  | EGFRwt | 3.01±1.09 | 3.21±1.15 | 3.23±1.17 | **-** | **-** | **-** |
|  | DNA | MLPA | EGFRwt | 1.72±0.24 | 1.87±0.03 | 1.89±0.06 | **-** | **-** | **-** |
|  |  |  | Total EGFR | 0.37±0.05 | 1.87±0.03 | 1.9±0.06 | **-** | **-** | **-** |
|  |  |  | CDKN2a | - | 0.99±0.02 | 1.01±0.03 | **-** | **-** | **-** |
|  |  |  | PTEN | 1.25±0.17 | 0.56±0.01 | 0.56±0.02 | **-** | **-** | **-** |
|  |  |  | other changes | MDM2 (0.27±0.04);  NFKB1A (2.1±0.29) | not detected | not detected | **-** | **-** | **-** |
|  | DNA | Sanger sequencing/IHC | IDH1 status | wt | - | - | **-** | **-** | **-** |
|  |  |  | TP53 status | p.Y205H | - | - | **-** | **-** | **-** |
|  | cells | FISH | EGFR amp/polysomy of chromosome 7 | - | polysomy, single cells with amp | - | **-** | **-** | **-** |
| **GB8** | **Source** | **Analysis** | |  | **p. 1** | **-** | **-** | **-** | **-** |
|  | mRNA | RT Real-time  PCR | EGFRwt expression | 29.19±5.31 | 27.89±7.01 | - | **-** | **-** | **-** |
|  |  |  | EGFRvIII expression | 37.93±7.42 | 16.5±3.91 | - | **-** | **-** | **-** |
|  | DNA | Real-time  PCR | Total EGFR | 352.12±19.7 | 27.37±5.29 | - | **-** | **-** | **-** |
|  |  |  | EGFRwt | 2.77±0.63 | 1.99±0.46 | - | **-** | **-** | **-** |
|  | DNA | MLPA | EGFRwt | 5.43±0.07 | 1.23±0.07 | - | **-** | **-** | **-** |
|  |  |  | EGFRvIII | 1.02±0.13 | 1.32±0.08 | - | **-** | **-** | **-** |
|  |  |  | CDKN2a | - | 0.84±0.05 | - | **-** | **-** | **-** |
|  |  |  | PTEN | 1.88±0.24 | 0.93±0.06 | - | **-** | **-** | **-** |
|  |  |  | other changes | MDM2 (0.26±0.03);  NFKB1A (2.1±0.27) | not detected | - | **-** | **-** | **-** |
|  | DNA | Sanger sequencing/IHC | IDH1 status | wt | - | - | **-** | **-** | **-** |
|  |  |  | TP53 status | wt | - | - | **-** | **-** | **-** |
|  | cells | FISH | EGFR amp/polysomy of chromosome 7 | - | single cells with amp | - | **-** | **-** | **-** |
| **GB9** | **Source** | **Analysis** | |  | **p. 1** | **p. 3** | **p. 9** | **p. 3** | **-** |
|  | mRNA | RT Real-time  PCR | EGFRwt expression | 2.11±0.35 | 1.15±0.25 | 0.95±0.19 | 2.21±0.26 | 2.16±0.25 | **-** |
|  |  |  | EGFRvIII expression | 96.71±2.3 | 18.43±2.54 | 8.34±0.52 | 0 | 5.82±0.67 | **-** |
|  | DNA | Real-time PCR | Total EGFR | 2.17±0.19 | 2.35±0.33 | 2.14±0.07 | 2.95±1.04 | 3.11±0.74 | **-** |
|  |  |  | EGFRwt | 2.78±0.05 | 1.99±0.09 | 1.78±0.5 | 1.27±0.63 | 3.16±1.1 | **-** |
|  | DNA | MLPA | EGFRwt | 1.53±0.19 | 1.63±0.06 | 1.37±0.13 | 0.97±0.04 | 1.37±0.08 | **-** |
|  |  |  | EGFRvIII | 1.62±0.21 | 15.75±0.58 | 2.47±0.23 | 0.99±0.04 | 18.29±1.05 | **-** |
|  |  |  | CDKN2a | 0.17±0.02 | 0 | 0.63±0.06 | 0.86±0.03 | 0 | **-** |
|  |  |  | PTEN | 0.68±0.09 | 0.58±0.02 | 0.78±0.07 | 0.90±0.03 | 0.83±0.07 | **-** |
|  |  |  | other changes | MDM2 (1.55±0.2);  CDK4 (1.31±0.17) | not detected | MDM2 (1.54±0.14);  CDK4 (1.71±0.16) | not detected | MDM2 (1.35±0.08);  CDK4 (1.41±0.08) | **-** |
|  | DNA | Sanger sequencing/IHC | IDH1 status | wt | - | - | - | - | **-** |
|  |  |  | TP53 status | p.Y234* | - | - | - | - | **-** |
|  | cells | FISH | EGFR amp/polysomy of chromosome 7 | - | single cells with amp | - | - | - | **-** |
| **GB10**  **stabilized** | **Source** | **Analysis** | |  | **p.1** | **p.7** | **-** | **p.5** | **p.7** |
|  | mRNA | RT Real-time PCR | EGFRwt expression | 26.84±1.05 | 26.11±1.07 | 17.63±0.52 | - | 16.69±1.01 | 12.48±0.79 |
|  |  |  | EGFRvIII expression | 6.38±0.14 | 4.76±0.17 | 0.38±0.08 | - | 1.45±0.15 | 1.31±0.19 |
|  | DNA | Real-time PCR | Total EGFR | 230.5±11.32 | 201.0±7.28 | 149.1±8.18 | - | 151.8±3.18 | 75.2±5.69 |
|  |  |  | EGFRwt | 115.6±4.56 | 93.2±2.45 | 78.2±3.25 | - | 66.27±8.22 | 50.5±4.43 |
|  | DNA | MLPA | EGFRwt | **-** | 13.9±0.46 | 15.43±1.65 | - | 11.68±1.62 | - |
|  |  |  | EGFRvIII | 22.43±3.5 | 16.34±0.54 | 19.49±2.09 | - | 13.75±1.35 | 9.43±1.16 |
|  |  |  | CDKN2a | - | 1.02±0.03 | 0.8±0.09 | - | 0.61±0.06 | - |
|  |  |  | PTEN | - | 0.93±0.03 | 0.61±0.06 | - | 0.56±0.05 | - |
|  |  |  | other changes | CDK4 (8.95±1.39);  PDGFRA (2.02±0.3); MDM2 (24.32±3.8); RET (0.53±0.08);  BRAF (1.53±0.24) | CDK4 (4.28±0.14);  MDM2 (22.31±0.73) | CDK4 (10.5±1.12);  MDM2 (33.38±3.6) | - | CDK4 (10.96±1.1);  MDM2 (29.74±2.9) | CDK4 (8.76±1.02); MDM2 (13.13±1.6);  RET (0.51±0.06) |
|  | DNA | Sanger sequencing/IHC | IDH1 status | wt | - | - | - | - | - |
|  |  |  | TP53 status | wt | - | - | - | - | - |
|  | cells | FISH | EGFR amp/polysomy of chromosome 7 | - | amp | - | - | - | - |
