# Supplementary material for: A way to understand idiopathic senescence and apoptosis in primary glioblastoma cells – possible approaches to circumvent these phenomena
Source: BMC Cancer. 2019 Sep 14;19:923. doi: 10.1186/s12885-019-6130-2 (PMC6744717; doi:10.1186/s12885-019-6130-2)
Supplement: Supplementary file 4 — Additional file 4: Table S4. The results of paired Student’s t-test for the comparison of cell biology features of neoplastic and normal cells in glioblastoma primary cultures in different conditions. (DOCX 17 kb) [file 12885_2019_6130_MOESM4_ESM.docx]

**Table S4.**The results of paired Student’s t-test for the comparison of cell biology features of neoplastic and normal cells in glioblastoma primary cultures in different conditions.

|  |  | **Cancer cells vs. normal cells** | | | **Normal cells** |
| --- | --- | --- | --- | --- | --- |
| **Primary cell line** | | monolayer | | NSC-like  early passage | monolayer  between passages |
|  |  | early passage | late passage |  |  |
| **GB6** | apoptotic | p = 0.010 | p = 0.037 | p = 0.019 | p = 0.051 |
|  | senescent | p = 0.015 | p = 0.015 | p = 0.009 | p = 0.113 |
|  | BrdU positive | p = 0.139 | p = 0.005 | p = 0.037 | p = 0.252 |
|  | others | p = 0.093 | p = 0.002 | p = 0.001 | p = 0.433 |
| **GB7** | apoptotic | p = 0.001 | p = 0.055 | p = 0.003 | p = 0.162 |
|  | senescent | p = 0.033 | p = 0.007 | p = 0.002 | p = 0.018 |
|  | BrdU positive | p = 0.010 | p = 0.003 | p = 0.021 | p = 0.113 |
|  | others | p = 0.007 | p = 0.483 | p < 0.001 | p = 0.414 |
| **GB8** | apoptotic | p = 0.074 | p = 0.087 | p = 0.037 | p = 0.213 |
|  | senescent | p = 0.056 | p = 0.005 | p = 0.010 | p = 0.500 |
|  | BrdU positive | p < 0.001 | p = 0.011 | p = 0.139 | p = 0.113 |
|  | others | p = 0.093 | p = 0.010 | p = 0.003 | p = 0.175 |
| **GB9** | apoptotic | N/A | p = 0.239 | p = 0.023 | p = 0.037 |
|  | senescent | p = 0.033 | p = 0.031 | p = 0.171 | p = 0.311 |
|  | BrdU positive | p = 0.014 | p = 0.013 | p = 0.027 | p = 0.037 |
|  | others | p = 0.500 | p = 0.162 | p = 0.024 | p = 0.193 |
