# Supplementary material for: A way to understand idiopathic senescence and apoptosis in primary glioblastoma cells – possible approaches to circumvent these phenomena
Source: BMC Cancer. 2019 Sep 14;19:923. doi: 10.1186/s12885-019-6130-2 (PMC6744717; doi:10.1186/s12885-019-6130-2)
Supplement: Supplementary file 5 — Additional file 5: Figure S5. Apoptosis of glioblastoma cells. Representative images showing classical apoptotic nuclei with TP53 accumulation (A) as well as activity of the synthetic Caspase 3/7 reporter in early passages of GB9. The number of Caspase 3/7 positive cells was higher in NSC-like conditions (C) than in monolayer (B) (quantitative data are shown in Fig. 3g-k and Additional file 4: Table S4). (DOCX 713 kb) [file 12885_2019_6130_MOESM5_ESM.docx]

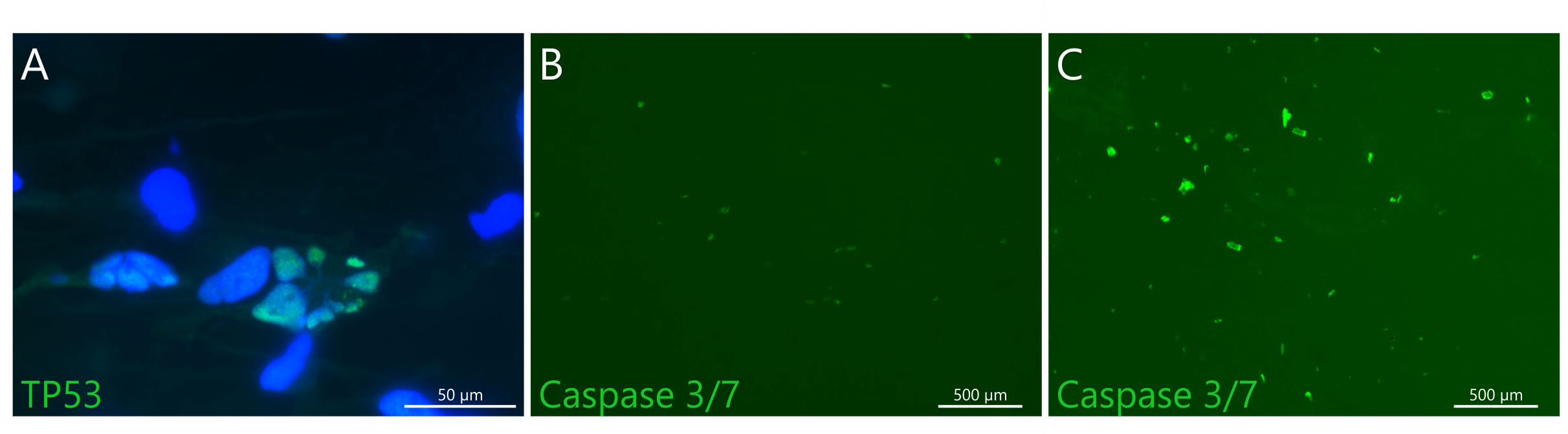


**Figure S1.** Apoptosis of glioblastoma cells. Representative images showing classical apoptotic nuclei with TP53 accumulation (A) as well as activity of the synthetic Caspase 3/7 reporter in early passages of GB9. The number of Caspase 3/7 positive cells was higher in NSC-like conditions (C) than in monolayer (B) (quantitative data are shown in Figure 3G-K and Suppl. Tab. 4).
